# Improved Photodynamic Therapy of Hepatocellular Carcinoma via Surface-Modified Protein Nanoparticles

**DOI:** 10.3390/pharmaceutics17030370

**Published:** 2025-03-14

**Authors:** Ahmed M. Abdelsalam, Amir Balash, Shaimaa M. Khedr, Muhammad Umair Amin, Konrad H. Engelhardt, Eduard Preis, Udo Bakowsky

**Affiliations:** 1 Department of Pharmaceutics and Biopharmaceutics, University of Marburg, Robert-Koch Straße 4, 35037 Marburg, Germany; ahmed.abdelsalam@pharmazie.uni-marburg.de (A.M.A.); umairami@staff.uni-marburg.de (M.U.A.); konrad.engelhardt@pharmazie.uni-marburg.de (K.H.E.); eduard.preis@pharmazie.uni-marburg.de (E.P.); 2 Department of Pharmaceutics and Pharmaceutical Technology, Faculty of Pharmacy, Al-Azhar University, Assiut 71524, Egypt; 3 Department of Pharmaceutical Chemistry, University of Marburg, Marbacher Weg 10, 35032 Marburg, Germany; amir.balash@pharmazie.uni-marburg.de; 4 Pharmaceutical and Fermentation Industries Development Center (PFIDC), City of Scientific Research and Technology Applications (SRTA-City), New Borg El Arab 21111, Egypt; vetshaimaa@gmail.com

**Keywords:** glycyrrhetinic acid, photosensitizer, PEGylation, parietin, active targeting, HepG2, targeted drug delivery, zein

## Abstract

**Background:** Photodynamic therapy (PDT) has evolved as a reliable therapeutic modality for cancer. However, the broad application of the technique is still limited because of poor bioavailability and the non-selective distribution of photosensitizers within host tissues. Herein, zein, a natural corn protein, was functionalized with glycyrrhetinic acid (GA) and polyethylene glycol (Z-PEG-GA) as a targeting platform for liver cancer cells. Parietin, as novel photosensitizer, was successfully encapsulated into zein via nanoprecipitation and used for the therapy of hepatocellular carcinoma. **Methods:** The in vitro phototoxicity of Z-PEG-GA nanoparticles and their non-functionalized control (Z-PEG) were assessed against hepatocellular carcinoma (HepG2 cells) and the In vivo biodistribution was determined in an adult male CD-1 Swiss albino mice model. **Results:** The formulated Z-PEG and Z-PEG-GA showed spherical shapes with average sizes of 82.8 and 94.7 nm for unloaded nanoparticles, respectively, and 109.7 and 111.5 nm for loaded nanoparticles carrying more than 70% of parietin, and Quantum yield measurements show that parietin’s photodynamic potential is conserved. Moreover, parietin-loaded Z-PEG-GA exhibited three-fold higher toxicity against liver cancer cells than its non-functionalized control and attained more than an eleven-fold enhancement in the generated intracellular reactive oxygen species (ROS) at a 9 J/cm^2^ radiant exposure. The generated intracellular ROS led to mitochondrial disruption and the release of cytochrome c. In vivo biodistribution studies revealed that fluorescence signals of Z-PEG-GA can persist in the excised animal liver for up to 24 h post-administration. **Conclusions:** Consequently, tailored zein can hold great potential for delivering several hydrophobic photosensitizers in anticancer PDT.

## 1. Introduction

Photodynamic therapy (PDT) is known as a minimally invasive therapeutic approach widely implemented to treat different cancer types in clinics [1,2]. The PDT concept relies on transferring photons of light to photoactive molecules termed photosensitizers (PSs) at specific wavelengths to produce highly reactive oxygen species (ROS), such as moieties of singlet oxygen and superoxide radicals [3,4,5]. The technique has been proven efficient compared to traditional chemotherapy as the generated ROS can induce apoptosis to malignant cells via different mechanisms, limiting the possibility of drug resistance [6]. Moreover, the poor vascularization of malignant cells causes the accumulation of PSs within cancerous cells more than in healthy tissues [7,8], which enables the application of PDT to control localized tumors in the bladder [9], prostate [10], and bile ducts using endoscopic optical fibers after the systemic infusion of PSs [2].

Several generations of PSs have already been implemented in cancer treatment. However, their side effects, such as poor bioavailability, photosensitivity, and hydrophobicity, have urged the need for the development of novel PSs [11]. One appealing approach is to utilize PSs of natural origin, e.g., parietin. Parietin is an anthraquinone derived from lichens, plants, and fungi as a secondary metabolite [12,13]. Parietin has been utilized as an antimicrobial and anticancer agent [14,15,16]. In addition, parietin has been successfully used as a PS [17]. Nevertheless, its use still suffers from its hydrophobicity, limiting its applicability as PS. An efficient approach implemented to improve the bioavailability and clinical application of PS is to incorporate them within smart nanoformulations [18,19,20,21]. Therefore, parietin was chosen in our study as a model hydrophobic natural PS against hepatocellular carcinoma cells (HepG2 cells).

Rekindled interest was given to plant-derived or rather natural-based nanocarriers in drug delivery applications due to their availability at a considerably cheap cost and the ease of fabrication into nanomaterials [22]. Zein, a renewable material, is one of the readily available proteins and the main prolamin fraction in maize. It is known for its biocompatibility and reliability in pharmaceutical applications, making it a sustainable option for various uses [22,23]. The amphiphilic properties of zein allowed for its utilization to fabricate nano-delivery platforms to improve the bioavailability of hydrophobic compounds such as PSs [24]. Lately, zein-based nanoparticulate platforms have been investigated for delivering the hydrophobic PS hypericin to eradicate hepatocellular carcinoma [25]. Hypericin-encapsulated zein showed two-fold higher toxicity in hepatocellular carcinoma cells than free PS [25]. Moreover, encapsulation into zein nanoparticles was found to conserve the photostability of indocyanine green (ICG) without loss of its photodynamic activity [26]. Hence, zein-based platforms are considered promising tools for the enhanced delivery of PSs to several biological targets.

The employment of a targeting strategy is of great interest in PDT. In the literature, three main approaches are usually described: passive, active, and stimuli-responsive targeting. Whereas passive targeting mainly relies on particle size and circulation time, stimuli-responsive targeting exploits internal conditions (e.g., pH differences, overexpressed enzymes) or depends on external factors, like heat, ultrasound, or magnetic field. In contrast, active targeting uses, among others, antibodies, peptides or small molecules that bind to cell-specific structures and mediate cellular uptake [27]. Targeted delivery to the liver was attempted using specific ligands interacting with the various receptors overexpressed on liver cells, e.g., folate, asialoglycoprotein, transferrin, hyaluronic acid, epidermal growth factor receptor, and others. Such approaches reduce the risk of PDT’s side effects like photosensitivity, likely to occur with most known PSs currently in use [28]. Moreover, active targeting can limit cellular filtering mechanisms such as the ABC pump (ATP-binding cassette), which is responsible for the efflux of PSs outside the cells [29,30]. In this context, zein was found to be very useful due to the feasibility of tailoring its surface with distinct targeting ligands, providing an extra option for delivering PSs to specific targets [31,32].

Among all targeting ligands, small molecules are the easiest and most cost-effective option, providing sufficient physiological stability, low immunogenicity, and rapid receptor-mediated uptake. The natural licorice hydrolysate extract glycyrrhetinic acid (GA) was found to interact specifically with rat liver membrane through a protein kinase C receptor lately denoted as GA-binding receptor [33], which recently became an attractive targeting ligand for the liver [34,35,36,37,38].

In our study, we wanted to combine GA as an active targeting entity with natural-based zein nanoparticles and test whether it would promote the anticancer activity of parietin to hepatocellular carcinoma and enhance zein’s in vivo liver targeting. Therefore, zein was PEGylated and tethered with glycyrrhetinic acid to form the Z-PEG or Z-PEG-GA, with the latter presumably being more hydrophobic because of the higher encapsulation efficiency of parietin. Following the synthesis, we prepared Z-PEG or Z-PEG-GA nanoparticles via nanoprecipitation and analyzed their physicochemical properties and biological activity.

## 2. Materials and Methods

### 2.1. Materials

α-zein (88–96% protein), tertiary butyl hydroperoxide (tbHP), fluorescamine, 5 aminofluorescine, dicyclocarbodiimid (DCC), and triethylamine were from Thermofisher scientific (Karlsruhe, Germany). Polyethylene glycol monomethyl ether 5 kd, N-hydroxy succinimide (NHS), 2′,7′-dichlorofluorescene diacetate (H_2_DCFDA), tetramethylrhodamine ethyl ester (TMRE), and 3-(4,5-dimethylthiazol-2yl)-2,5-diphenyltetrazolium bromide (MTT) were purchased from Sigma Aldrich (Darmstadt, Germany). O-(2-Aminoethyl)-O′-(2-carboxyethyl)-polyethylene glycol 5000-hydrochloride (mono mePEG) was obtained from Rapp Polymere (Tübingen, Germany). Glycyrrhetinic acid was procured from abcr GmbH (Karlsruhe, Germany). Carbonyl cyanide 4-(trifluoromethoxy) phenylhydrazone (FCCP) and parietin were provided by Cayman Chemical (Ann Arbor, MI, USA).

### 2.2. Synthesis and Characterization of Zein–PEG

PEGylated zein was synthesized and characterized following our previously reported procedures [25]. The PEGylation process started by first dissolving purified zein powder and mono mePEG in anhydrous dimethyl sulfoxide 1:1 (*w*/*w*). A total of 0.5 mL of triethylamine was then introduced as a catalyst. The reaction vessel was incubated at room temperature for 72 h to allow sufficient time for PEGylation. The product was purified via dialysis against pure water for 48 h. The water was replenished every 6 h. Finally, PEGylated zein was freeze-dried and kept at 4 °C until it was needed for experiments. Spectroscopic techniques like FT-IR and proton NMR were applied to characterize the chemical structure of the modified zein. High-performance size exclusion chromatography multiangle light scattering (HP-SEC-MALS) determined the molar mass distribution.

### 2.3. Synthesis of Glycyrrhetinic Acid–PEG–Zein Conjugate

#### 2.3.1. Synthesis of Glycyrrhetinic Acid Succinimidyl Ester

The terminal carboxylic acid group of GA was activated for further binding to the PEG chain using the N-hydroxysuccinimide/dicyclocarbodiimid (NHS/DCC) coupling chemistry. A total of 1 g (2.124 mmol) of glycyrrhetinic acid was utterly dissolved in 20 mL of degassed tetrahydrofuran, followed by the addition of DCC (0.44 g, 2.124 mmol, 1 mol equivalent), and the mixture was stirred for 30 min in an ice bath. Then, 0.25 g of NHS (2.124 mmol, 1 molar equivalent) was added to the cold mixture. The reaction was kept stirring under nitrogen for 24 h in the dark to prevent the decomposition of glycyrrhetinic acid. The mixture was filtered to remove the precipitated side product (dicyclohexyl urea), while excess organic solvents were evaporated to obtain the product, which was further redissolved in tetrahydrofuran, filtered, and rotary-evaporated to obtain the activated glycyrrhetinic acid. The product was thoroughly washed with ethanol and diethyl ether, and organic solvents were removed using a desiccator. The produced active acid was traced by thin-layer chromatography (TLC) using a chloroform/methanol 9:1 (*v*/*v*) mixture as eluent.

#### 2.3.2. Synthesis of Carboxy-PEG-GA Conjugate

In a 25 mL round-bottomed flask, α-carboxy-ω-amine PEG (5.311 kDa, 0.1 g, 17 mmol) was dissolved in 10 mL of dry methylene chloride under a stream of nitrogen gas. Glycyrrhetinic acid-N-hydroxy succinimide ester (GA-NHS) (0.021 g, 2 mol equivalent) was added, followed by 200 µL of triethylamine. The reaction was left under nitrogen. Stirring was continued for 48 h in the dark while monitoring the reaction through TLC using chloroform/methanol 5:1 (*v*/*v*) as an elution system. At the end of the reaction period, the methylene chloride was rotary-evaporated. The residue was further washed with ethanol (96% (*v*/*v*)) to dissolve the conjugated PEG, leaving the reaction byproducts as sediment. The mixture was filtered, and the pale green filtrate was purified by running over the Sephadex LH 20 column using ethanol/DCM 70:30 *v*/*v*. The product was rotary-evaporated, redissolved in methylene chloride, and precipitated by dropping into cold diethyl ether. The precipitated PEG-GA was filtered through Wattman filter paper and dried in a desiccator. The conjugation percentage of GA to PEG was indirectly quantified using fluorescamine spectrofluorometric assay (Supplementary Materials, Section S1.1) [39].

#### 2.3.3. Synthesis of the Z-PEG-GA Conjugate

**Scheme 1:**
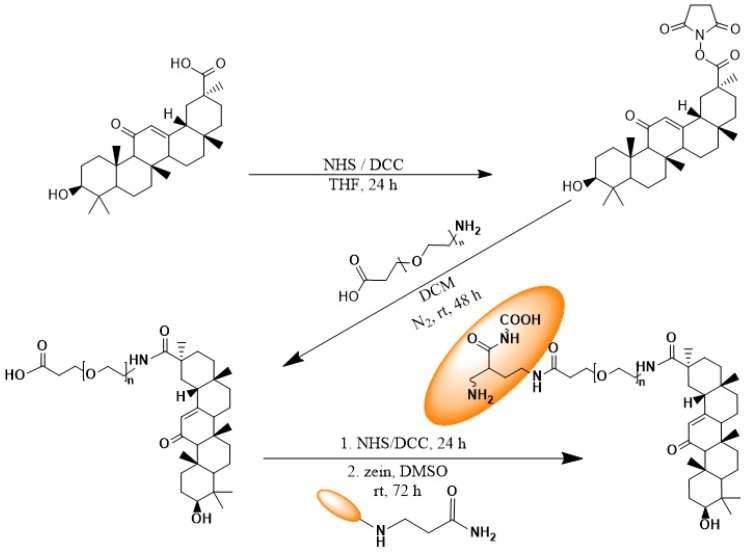
Chemical synthesis of glycyrrhetinic acid-tethered zein.

The conjugation of GA-PEG-carboxyl and zein was conducted in two consecutive steps employing NHS/DCC coupling chemistry. Firstly, the carboxyl functionality was activated by dissolving carboxy-PEG-GA (0.08 g, 14 mmol) in 20 mL of nitrogen-flushed methylene chloride in a 50 mL round-bottom flask, followed by the addition of DCC (0.007 g, 2 mol equivalent) and NHS (0.004 g, 2 mol equivalent). The reaction was left stirring for 24 h under a nitrogen atmosphere. After the reaction period, the mixture was filtered to remove byproducts, while the filtrate was rotary-evaporated to yield the reactive NHS ester as a pale green residue. Secondly, 3 mL of nitrogen-flushed DMSO was added to the residue, followed by 0.08 g of purified zein and 100 µL of triethylamine. The reaction was then left under nitrogen for 72 h. The Z-PEG-GA was further acquired by dialyzing the mixture against distilled water for 48 h. Water was changed every 6 h to obliterate excess unreacted PEG. The dialyzed colloidal product of the Z-PEG-GA was lyophilized to give a pale-yellow fluffy powder. The steps used in the whole synthesis of the Z-PEG-GA platform are summarized in Scheme 1.

### 2.4. FT-IR and H^1^NMR Spectroscopy

FT-IR analysis was conducted using a Brucker FT-IR instrument (Bruker Optic. GmbH, Ettlingen, Germany). FT-IR measurements of the neat solid samples were recorded within the wavenumber range from 4000 to 400 cm^−1^. For the ^1^H NMR analysis, the test candidate was dissolved in an appropriate NMR solvent in a definite amount, and the samples were further analyzed using an auto-tune sample head Nuclear Magnetic Resonance JEOL ECX-400 Instrument (Japan, Tokyo).

### 2.5. Preparation and Characterization of Parietin-Loaded Z-PEG-GA Nanoparticles

#### 2.5.1. Preparation of Z-PEG and Z-PEG-GA Nanoparticles Loaded with Parietin

Z-PEG and Z-PEG-GA nanoparticles were synthesized via the solvent injection technique. Briefly, 3 mL of Z-PEG or Z-PEG-GA (PEGylated zein dissolved in 90% ethanol at a concentration of 0.5% (*w*/*v*)) was introduced into 7 mL of citrate buffer with a pH of 7.44 [40]. The mixture was subjected to stirring for 48 h at room temperature (700 rpm) to ensure the complete evaporation of ethanol while maintaining a constant volume of 7 mL with citrate buffer. To formulate nanoparticles loaded with parietin, either the Z-PEG or Z-PEG-GA solution was combined with parietin (0.25 mg dissolved in pure ethanol) and left to stir overnight in the absence of light before being added to the buffer. The prepared formulations were freeze-dried and stored at −20 °C for further characterization.

#### 2.5.2. Determination of Encapsulation and Loading Efficiency of Parietin

The quantification of parietin encapsulation within zein nanoparticles was performed directly. In brief, 1 mL of zein nanoparticles underwent centrifugation at 19.78 × 10^3^× *g* for 20 min, utilizing a Hettich centrifuge (Andreas Hettich GmbH, Tuttligen, Germany). The resulting pellets were then subjected to digestion with 90% *v*/*v* ethanol. Subsequently, the amount of entrapped parietin was estimated by UV/V is spectrophotometry (Shimadzu-UV mini 1240, Shimadzu Deutschland GmbH, Germany, Duisburg) at 434 nm, employing a pre-established parietin calibration curve in ethanol. The percentage of encapsulated parietin was computed using Equation (1) as follows:

(1) $$\%EE=\frac{amount\;of\;parietin\;in\;pellets}{total\;amount\;of\;parietin}\times 100\%$$

The loading efficiency was evaluated after digesting a definite mass of the freeze-dried formulation with 90% ethanol solution. The percentage of loaded parietin was then estimated using Equation (2).

(2) $$\%DL=\frac{weight\;of\;loaded\;parietin}{total\;weight\;of\;the\;formulation}\times 100\%$$

#### 2.5.3. Dynamic Light Scattering (DLS)

The hydrodynamic diameter, polydispersity index (PDI), and zeta potential of zein nanoparticles were evaluated by means of Zetasizer Nano ZS instrument (Malvern Panalytical GmbH, Kassel, Germany). Each sample, consisting of 100 µL, was diluted with ultra-pure water in a 1:10 (*v*/*v*) ratio to reach a final volume of 1 mL. Dynamic Light Scattering (DLS) measurements were carried out at room temperature, considering a minimum of three independent measurements for each sample.

#### 2.5.4. Atomic Force Microscopy (AFM)

For the standard AFM measurement, a 1:100 (*v*/*v*) diluted sample dispersion in ultrapure water (50 µL) was carefully pipetted onto a silicon wafer and left to settle for 15 min. The sample was naturally dried at room temperature. The AFM analysis was conducted utilizing the NanoWizard^®^-3 NanoScience AFM system (JPK BioAFM, Bruker Nano GmbH, Berlin, Germany) equipped with a vibration-damped setup (i4 Series—Active Vibration Isolation, Accurion GmbH, Göttingen, Germany). A commercial cantilever was used (HQ:NSC14/Al BS, Mikromasch Europe, Wetzlar, Germany), featuring a resonance frequency of 160 kHz and 5 N/m nominal force constant. The scanning speed ranged from 0.5 to 1.5 Hz, while AC mode was employed for measurements in air. Both height- and amplitude-measured modes were used to visualize the acquired images, and subsequently, the raw images were processed using the JPK data processing software (JPK, Berlin, Germany, JPK Data Processing Software Version 4.2).

#### 2.5.5. Release Study of Parietin

The freeze-dried parietin-loaded zein formulations, with a final parietin concentration of 200 µg/mL, were gently dispersed by shaking in 5 mL dark glass vials. A total of 5 mL of 0.1 M PBS with a pH of 7.4 and containing 1% *w*/*w* tween 80 was used as the release medium to conserve sink conditions. The vials were placed on a thermocontrolled shaker (EB, Johannes Otto Gmbh, Germany) at 37 °C and 100 rpm. At predetermined time intervals (1, 2, 3, 4, 5, 6, 7, 8, 12, 24, 48, and 72 h), 1 mL of the release medium was removed and centrifuged for 5 min at 8.83 × 10^3^ g. Afterward, the supernatant was withdrawn, and the cumulative percentage of released parietin in the withdrawn medium was estimated using UV/Vis spectrophotometry (Shimadzu UV mini-1240, Shimadzu Deutschland GmbH, Duisburg, Germany) at 438 nm and a calibration curve for parietin in PBS with 1% *w*/*w* tween 80. Each formulation’s release study was conducted in triplicate. The resulting release data were subjected to mathematical analysis, fitting into the zero-order model Equation (3), first-order model Equation (4), and the Higuchi diffusion Equation (5). This analysis aimed to identify the most suitable kinetic model for the release of the hydrophobic drug from the zein platforms.

(3) $$Q_{t}\;=\;Q_{0}\;-\;kt\;$$

(4) $$\log Q_{t}\;=\;\log Q_{0}-\frac{kt}{2.303}$$

(5) $$Q\;=kt^{\frac{1}{2}}$$

Q_o_ and Q_t_ are the initial and the released amounts of parietin, respectively; k is the diffusion rate constant. Q is the released amount of parietin per unit surface area.

#### 2.5.6. Assay of Singlet Molecular Oxygen (^1^O_2_)

The ability of the free and formulated parietin to generate singlet oxygen was examined according to some reported methods [41,42]. We employed uric acid as a singlet molecular oxygen quencher instead of other quenchers, such as DPBF, that degrade in the blue light region [42,43,44]. Uric acid (1 mM solution) was prepared in 0.1 M PBS and mixed with unformulated parietin or zein-parietin nanoparticles in 1.5 mL Eppendorf tubes. The final parietin and uric acid concentrations were fixed to 2.9 × 10^−5^ M and 28.1 × 10^−5^ M, respectively. Samples were pipetted into a 96-well plate (Nunc, Thermo Fisher Scientific GmbH, Dreieich, Germany), and the plate was irradiated using a blue LED at 457 nm and irradiance of 220 mW/cm^2^ for changing time intervals (1, 2, 3, 4, 5, 6, and 7 min). The decline in the uric acid absorption peak at 291 nm was recorded via a UV/Vis spectrophotometer (Thermo Fisher Scientific GmbH, Dreieich, Germany). The absorbance of parietin solution or parietin–zein nanoparticles was recorded simultaneously. The relative quantum yields of the free and zein-loaded parietin nanoparticles were calculated in accordance with rose Bengal as a known reference PS by applying Equation (6):

(6) $${ɸ}_{\Delta }^{S}={ɸ}_{\Delta \;}^{R}\times \;\frac{k_{S}}{k_{R}}\times \frac{1-10_{R}^{-OD}}{1-10_{S}^{-OD}}$$

k_S_ and k_R_ are the depletion rate constants for the sample and reference PS, respectively. OD is the optical density of the reference and sample measured at 457 nm, and ${ɸ}_{\Delta }^{R}$ is the quantum yield of the standard PS (rose Bengal).

#### 2.5.7. Stability of Zein Nanoparticles with Serum

The stability of zein nanoparticles in 10% *w*/*v* bovine serum solution was established according to our previously reported procedures. In brief, 700 µL of each zein formulation was mixed with the bovine serum solution in a 1:1 ratio. The resulting mixture was then vortexed and kept in a thermostatic shaker (IKA, Staufen im Breisgau, Germany) at fixed temperature (37 °C) and shaking speed (100 rpm). The stability of zein formulations was investigated by monitoring alterations of nanoparticle’s diameter, PDI, and ζ-Potential at varying incubation intervals (1, 3, 6, 12, 24, and 48 h). Freshly produced samples were used as control for comparison.

### 2.6. In Vitro Biological Investigations

#### 2.6.1. Cell Culture

The HepG2 cell line, which represents hepatocellular carcinoma, was acquired from ATCC (American Type Cell Culture, Manassas, VA, USA). These cells were cultured in Dulbecco’s Modified Eagle Medium (DMEM), supplemented with 10% fetal calf serum and 1% non-essential amino acids from Capricorn Scientific (Ebsdorfergrund, Germany). The cultivation was carried out at 37 °C and 5% CO_2_ in a humidified environment. For the experiments, cells from passage numbers 10 to 30 were used, and they were appropriately split using trypsin/EDTA when needed.

#### 2.6.2. LED Device

This study employed a prototype light-emitting diode (LED) (Lumundus Gmbh, Eisenach, Germany) previously described in [45]. The LED features an array of low-power light-emitting diodes specifically tailored for multiwell plates emitting blue light at a maximum wavelength of 457 nm. Irradiance was measured for each selectable current (20, 40, 60, 80, or 100 mA). By choosing a current and irradiation time, the radiant exposure was defined.

#### 2.6.3. Cell Viability Assay

HepG2 cells were cultivated into individual wells of a 96-well plate, with 2 × 10^4^ cells per well. After 24 h, the cells were exposed to free parietin and parietin loaded into zein nanoparticles for a duration of 4 h. Following this incubation period, the medium was removed, and the cells were washed with buffered saline. Subsequently, the cells were subjected to blue LED irradiation at different radiant exposures (5 and 9 J/cm^2^). After the irradiation, the cells were further incubated for 24 h. To assess the number of surviving cells, the cells were treated with MTT at a concentration of 5 mg/mL in PBS. Briefly, 100 µL of the MTT reagent (diluted in culture medium at 10% *v*/*v*) was added to each well and incubated for 4 h. MTT/medium was removed, and 100 µL of DMSO was added to each well. The plates were then shaken for 15 min at 37 °C to dissolve the formazan crystals. The absorbance of the dissolved formazan was measured at 570 nm using a microplate reader (FLUOStar Optima, BMG Labtech Gmbh, Offenburg, Germany). The obtained results were analyzed by comparing the optical density of the treated cells with the optical density of untreated cells (negative control) and cells treated with 0.1% triton-X100 (positive control). Generally, cell viability was considered to be 100% in the control group. The percentage of cell survival was determined using Equation (7) as follows:

(7) $$\%\;Cell\;Viability=\frac{\;{OD}_{Sample}-{OD\;}_{Blank}}{\;{OD}_{Control}-{OD\;}_{Blank}}\times 100\%$$

OD_sample_ and OD_Control_ are the respective optical densities for the treated and the untreated control cells. OD_Blank_ is the absorbance of pure DMSO.

#### 2.6.4. Uptake Study in HepG2 Monolayer

HepG2 cells were cultured on coverslips in 12-well plates with a seeding density of 1 × 10^5^ cells per well. After 24 h of incubation to achieve confluency, cells were exposed to free parietin and parietin-loaded zein at a concentration equivalent to 1.7 µM of free parietin for 4 h. To serve as a control for the Z-PEG-GA formulation, HepG2 cells were pretreated with 20 µM of glycyrrhetinic acid for 2 h before being exposed to Z-PEG-GA. Subsequently, the cells were fixed with 4% paraformaldehyde for 20 min, washed with cold PBS containing calcium and magnesium, and stained with DAPI (0.6 µg/mL) for 10 min. Placing the coverslips on glass slides, they were protected with FluroSaveTM Reagent and subjected to cellular imaging through confocal microscopy (CLSM) with an LSM 700 system (Carl Zeiss, Göttingen, Germany). Furthermore, a separate uptake study utilized glycyrrhetinic acid (GA) conjugated to fluorescein isothiocyanate (FITC) (Supplementary Materials, Section S1.2) to track its passage in HepG2 cells over 30 min and 1 h. The acquired CLSM images were subjected to image analysis using the ImageJ analysis program (ImageJ Version 1.54, NIH, Bethesda, MD, USA).

#### 2.6.5. Intracellular ROS Assay

The generation of reactive oxygen species in HepG2 cells was investigated. Briefly, HepG2 cells were cultivated in a 96-well plate at a density of 2 × 10^4^ cells per well and incubated for 24 h. Following this, the cells were exposed to the respective IC_50_ concentrations of free parietin and parietin-loaded zein nanoparticles for 4 h. As a positive control, tbHP at a final concentration of 50 μM was utilized. After the removal of the medium, the cells were then washed with sterile PBS and treated with H_2_DCFDA at a final concentration of 10 μM. After a 30-min incubation, the cells underwent two PBS washes and were subsequently irradiated with 9 J/cm^2^ (fixed current of 100 mA and irradiance of 220 mW/cm^2^). Then, the PBS was removed, and the cells were trypsinized and lysed using 200 µL of cell lysis buffer consisting of NaOH (0.2 N) and Triton X-100 (0.5% *w*/*v*) while shaking at 37 °C and 150 rpm in a shaker incubator. After 15 min, the cell lysate was transported to a dark 96-well plate, and the fluorescence of the oxidized DCF was measured at excitation and emission wavelengths of 485 nm and 535 nm, respectively. A similar approach was followed for the non-irradiated cells. Finally, the fluorescence activity obtained from non-irradiated and irradiated cells was recorded within the same plate. The changes in cellular fluorescence activity before and after treatment were observed using fluorescence microscopy (Olympus CKX53, Tokyo, Japan).

#### 2.6.6. Mitochondrial Membrane Potential Assay (ΔΨm)

The mitochondrial potential was assessed in two separate ways using the TMRE and JC-1 fluorescent dyes. HepG2 cells were cultured in a 96-well plate with a seeding density of 2 × 10^4^ cells per well and incubated for 24 h. Following this, the medium was removed, and the cells were washed with PBS before treatment with an IC_50_ equivalent amount to free parietin and zein-loaded parietin formulations. After a 4-h incubation, the cells were washed twice with PBS, a new medium was added, and the cells were exposed to blue light LED irradiation at a radiant exposure of 9 J/cm^2^. Subsequently, 24 h later, the medium was replaced with a new medium containing TMRE dye (400 nM per well) for a 30-min incubation period. As negative and positive controls, untreated cells and FCCP-treated cells (100 µM per well) were included. The medium was discarded, and the cells were washed with PBS (1×) and then with 0.2% *w*/*v* BSA (3×) to remove any dye residuals. The plates were finally equilibrated for 15 min at room temperature, and the fluorescence was examined at excitation and emission wavelengths of 549 nm and 575 nm, respectively.

#### 2.6.7. Immunocytochemistry Analysis of Cytochrome C Release

Cytochrome c release from mitochondria following irradiation of cells was assessed by means of immunocytochemistry using an apoptosis ICC antibody assay kit (ab110417 ApoTrack^TM^, Mitosciences, Eugene, OR, USA) and cells were treated according to the protocol provided by Abcam. Briefly, HepG2 cells were seeded in a 12-well plate over coverslips at seeding density (1 × 10^5^ cells/well). Cells were cultivated for 24 h in DMEM medium. Cells were treated with an amount equivalent to the IC_50_ of the free and parietin-loaded zein formulation. After 4 h, cells were washed thrice with cold PBS, and a new medium was added. Afterward, cells were irradiated with blue LED with 9 J/cm^2^. Next, cells were fixed for 10 min with 4% paraformaldehyde (100 µL/well), washed twice with PBS, and permeabilized for 10 min with 0.1% tritone (500 µL/well) at room temperature. Cells were washed once with PBS and further incubated for 1 h at room temperature with 10% goat serum (500 µL/well) to block the cells. Cells were incubated overnight at 4 °C with two primary antibodies (Anti-Cytochrome C monoclonal Ab and the Anti-Complex Vα monoclonal Ab) in 10% goat serum at a final concentration of 2 µg/mL for each. On the next day, cells were washed 5–6 times with 1% goat serum and further treated in the dark with the two secondary antibodies (goat anti-mouse IgG2a-FITC and goat anti-mouse IgG2b-TXRD), 2 µg/mL each, in 10% goat serum for 1 h at room temperature. After incubation, cells were rinsed 5–6 times with 1% goat serum and further stained with DAPI (0.3 µg/mL) for 10 min. Finally, coverslips were washed with PBS and mounted over microscope glass slides using fluorosafe mounting liquid, and cells were further visualized through CLSM imaging.

### 2.7. In Vivo Biodistribution Study

#### 2.7.1. Animal Ethical Approval

The proposed drug delivery system uses GA as an active targeting entity. After thoroughly evaluating the improved activity of parietin-loaded zein nanoparticles in vitro, we tested the biodistribution of Z-PEG and Z-PEG-GA nanoparticles in adult male CD-1 Swiss albino mice to assess the amount of particles residing in the liver and other main organs (i.e., brain, heart, lung, viscera, spleen, kidney, uterus). All animal procedures were approved by the Institutional Animal Care and Use Committee (IACAUC) of the Pharmaceutical and Fermentation Industries Development Center (No. 43-12L-9021) and by the committee of the Egyptian Ministry of Scientific Research (No. 190, 2015) following NIH guidelines and the ARRIVE guidelines 2.0 for principles of laboratory animal care. Thirty-six adult male CD-1 Swiss albino mice, aged 6–8 weeks and weighing 22 ± 2 g, were purchased from the animal facility of the Faculty of Veterinary Medicine, Alexandria University, Egypt. No other exclusion criteria were defined. Data from all animals were included in this study. Each mouse was allocated a unique number, and they were randomized into two groups (each n = 18) via Microsoft Excel. The mice were housed in propylene cages in groups (6 per cage, total of 6 cages), fed standard rodent chow, and permitted ad libitum access to water. Standard environmental conditions were maintained (21 °C, 45–55% humidity and light/dark cycles 12:12 h). They were acclimated for 7 days prior to the start of the experiment. The groups were either treated with ICG-loaded Z-PEG (control; sample 1) or ICG-loaded Z-PEG-GA (sample 2) nanoparticles. All investigators directly involved in the execution (i.e., administration, anesthesia, euthanasia, imaging) were unaware of the samples’ composition. The sample size was chosen so that each time point (0.5, 2, 4, 6, 12, and 24 h) would include triplicates and proper statistical significance could be calculated. The mice were imaged under anesthesia using isoflurane inhalation (Forane—Abbott, Chicago, IL, USA). The mice were euthanized by placing them in an induction chamber continuously filled with 5% isoflurane in oxygen and keeping them there for at least 10 min after signs of deep anesthesia were detectable.

#### 2.7.2. Preparation of ICG-Loaded Z-PEG and Z-PEG-GA

ICG-loaded zein nanoparticles were prepared as follows. In brief, 60 mg of Z-PEG or Z-PEG-GA and 0.5 mg of ICG were dissolved in 3 mL of 90% *v*/*v* ethanol. The mixtures were stirred overnight in the dark and added dropwise into 7 mL of ultra-pure water under magnetic stirring. Twenty-four hours later, ICG nanoparticles were acquired as a pale green dispersion and eluted over the Sephadex G25 column to separate the unentrapped dye. The actual amount of entrapped ICG was determined by dissolving 1 mL of the separated dispersion with 90% *v*/*v* ethanol. UV absorption of samples was measured at 788 nm, and the entrapped amount was calculated using a previously established calibration curve of ICG in ethanol.

#### 2.7.3. In Vivo Optical Imaging

Lyophilized Z-PEG and Z-PEG-GA nanoparticles loaded with indocyanine green (ICG) were dispersed in PBS and injected into the tail vein (200 µL/mouse, containing 7 µg of ICG). Biodistribution of Z-PEG (control) or Z-PEG-GA nanoparticles was assessed by acquiring near-infrared fluorescence images of ICG at λ_ex_ = 730 nm and λ_em_ = 830 nm, using PhotonIMAGER™ OPTIMA (Biospace Lab, Paris, France). Image acquisition was fixed to 5 s, and the real-time NIR imaging was conducted at different time points (0.5, 2, 4, 6, 12, and 24 h). At each time point, animals were ethically euthanized, and the liver and other main organs (i.e., brain, heart, lung, viscera, spleen, kidney, uterus) were excised and analyzed with ex vivo imaging. Image analysis was processed using a built-in PhotoAcquisition M3Vision analysis software (LTF Labortechnik GmbH & Co. KG, Wasserburg, Germany, V2.1).

### 2.8. Statistical Analysis

Statistical analysis was carried out using GraphPad Prism 6.1 software (GraphPad Software Inc., Boston, MA, USA). Data were presented as the mean ± standard deviation (SD), and statistical significance between means was determined by employing one-way and two-way ANOVA. Statistical significance was expressed as nonsignificant (ns), * *p* < 0.05, ** *p* < 0.01, *** *p* < 0.001 and **** *p* < 0.0001.

## 3. Results

### 3.1. Synthesis and Characterization of Glycyrrhetinic Acid-Conjugated Zein

**Figure 1:**
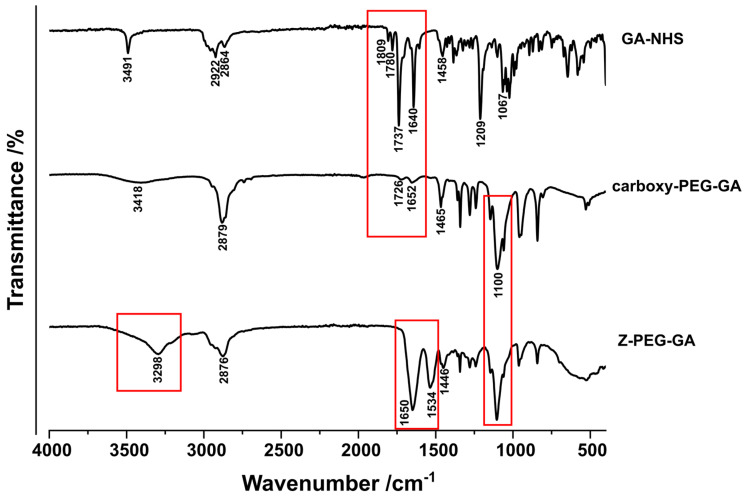
FT-IR spectra of the glycyrrhetinic acid succinimidyl ester, carboxy-PEG-GA, and Z-PEG-GA conjugate.

NHS/DCC coupling chemistry was employed to conjugate GA through a PEG spacer onto the surface of zein. The conjugation was first proved using FT-IR spectroscopy. Figure 1. illustrates the FT-IR investigations, showing that the glycyrrhetinic acid NHS ester peaks appeared at 1780 and 1809 cm^−1^, corresponding to the succinimidyl ester groups [46]. The succinimidyl peaks vanished after GA conjugation with PEG; moreover, the GA-related aliphatic double bonds (C=O and C=C stretching peaks) were conserved at 1726 cm^−1^ and 1652 cm^−1^. PEGylated zein showed the amide I peak at 1650 cm^−1^ and a shifted amide II peak at 1534 cm^−1^, indicating a successful Z-PEG coupling [25]. Additionally, the PEGylated zein showed peaks at 3298 cm^−1^ and 1100 cm^−1^ that might be ascribed to the amide N-H and the ethylene oxide C-O stretching, respectively.

**Figure 2:**
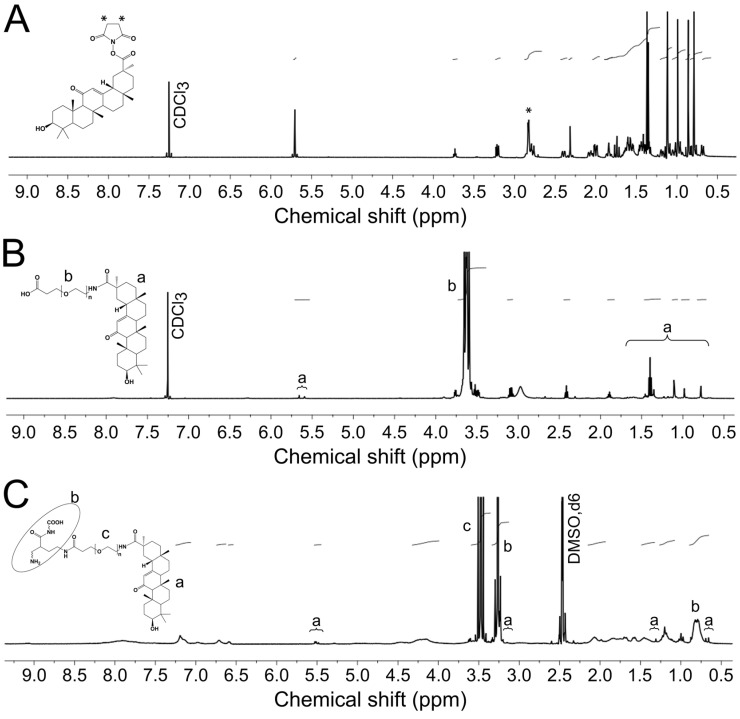
H^1^NMR spectra of glycyrrhetinic acid succinimidyl ester (**A**), carboxy-PEG-GA (**B**), and Z-PEG-GA (**C**) conjugates.

The conjugated Z-PEG-GA structure was additionally characterized with H^1^NMR spectroscopy (Figure 2). The glycyrrhetinic acid succinimidyl ester was confirmed by the triplet peak that appeared at 2.8 ppm, indicating the chemical shift of CH2-CH2 proton of the succinimide ester (Figure 2A). The conjugation of glycyrrhetinic acid to the PEG chain was indicated by the appearance of the peaks at 0.85, 1.1, 1.4, and 5.56 ppm related to the steroidal structure of glycyrrhetinic acid in addition to the ethylene oxide chain peaks at 3.6 ppm corresponding to the conjugated PEG chain (Figure 2B). The Z-PEG-GA conjugate was confirmed by the appearance of amide protons at the 3.3 ppm chemical shift, ethylene oxide at 3.4 ppm, and glycyrrhetinic acid protons at 0.58–1.4 ppm (Figure 2C).

Furthermore, the glycyrrhetinic acid coupling to the PEG chain, prior to conjugation to zein, was fluorometrically quantified using fluorescamine dye [39]. As depicted in Figure S1A, the interaction of the free PEG chain with fluorescamine brought about highly fluorescent conjugate at the corresponding excitation and emission intensities, in contrast, GA-coupled PEG showed minimal fluorescence at the same conditions, which was ascribed to the blockage of most of the free amine functionalities of the PEG molecules by GA. The GA-conjugated PEG exhibited 5-fold lower fluorescence than the free PEG-amine, as shown in Figure S1B. Quantification of the amount of conjugated GA was indirectly assessed by comparing the fluorescence intensity obtained by reacting 25 µM of free and GA conjugated PEG with fluorescamine via a fluorescamine–PEG calibration curve (Figure S1C). The outcomes indicated that more than 80% of the reacted GA was successfully coupled to the PEG chain.

### 3.2. Preparation and Characterization of Parietin-Loaded Zein Nanoparticles

Zein was PEGylated and tethered with glycyrrhetinic acid to form the Z-PEG or Z-PEG-GA platforms for liver targeting. Parietin was encapsulated into the two zein platforms via the nanoprecipitation method [25]. Table 1 illustrates the obtained DLS measurements, showing that the blank Z-PEG and Z-PEG-GA formulations obtained sizes of 82.8 ± 3.7 and 94.7 ± 6.6 nm, respectively. The acquired DLS data revealed sizes of 109.7 ± 16.9 and 111.5 ± 6.3 nm for the parietin-loaded Z-PEG and Z-PEG-GA formulations, respectively. The PDI values were around 0.19 and 0.24 for the blank and loaded Z-PEG, respectively. In addition, PDI of 0.25 and 0.19 were recorded for the blank and loaded Z-PEG-GA, respectively. Moreover, the entrapment and loading efficiencies of Z-PEG were 70.3 ± 11 and 1.11 ± 0.29 percentages, respectively. Comparatively, the Z-PEG-GA showed slightly higher values of 86.1 ± 7.1 and 1.29 ± 0.24 percentages for the entrapment and loading efficiencies, respectively, which might be attributed to the presence of glycyrrhetinic acid, which increases the hydrophobic character of the Z-PEG-GA-based nanoparticles. In contrast to other reported zein formulations, PEGylated zein was found to produce nanoparticles and micelles of relatively smaller sizes, acceptable PDI, higher stability, and elevated drug loading percentages [40].

**Figure 3:**
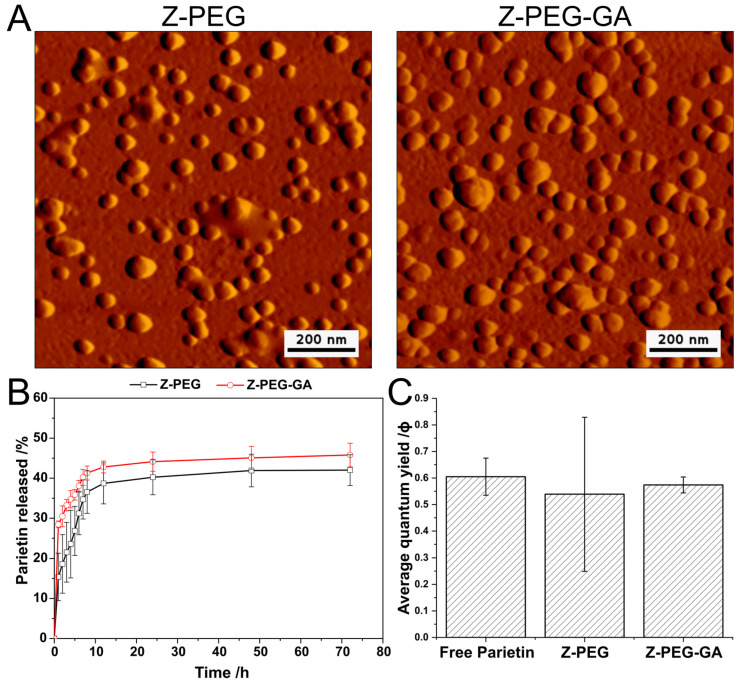
Amplitude AFM images of the Z-PEG and Z-PEG-GA formulations (**A**). Percentage of parietin release at different time intervals for 72 h in PBS pH 7.4 containing 1% *w*/*v* tween 80 (**B**). Average quantum yield values obtained from the degradation of uric acid in the presence of free parietin, Z-PEG, and Z-PEG-GA formulations after blue-light irradiation (**C**).

The morphological characteristics of the designed zein nanoparticles were assessed by atomic force microscopy (Figure 3A). Both the Z-PEG and Z-PEG-GA formulations manifested nearly spherical shapes with smooth surfaces. The average diameters of the nanoparticles were around 60 and 80 nm for the Z-PEG and HyZ-PEG-GA formulations, respectively, which is in good correlation with the hydrodynamic diameter obtained by DLS measurements.

The assessment of parietin release from the PEGylated zein platforms at pH 7.4 indicated that both the Z-PEG and Z-PEG-GA formulations manifested a burst release pattern in the first 8 h, followed by a slower release cascade up to 72 h (Figure 3B). The percentages of parietin released after 8 h were 36.5 ± 5.3 and 41.3 ± 1.7 for Z-PEG and Z-PEG-GA, respectively. However, after 72 h, the percentages of parietin released were increased to 42 ± 3.8 and 45.8 ± 2.9, respectively, indicating no significant release difference between the two formulations. PEG coats were therefore proven to reduce the hydrophobicity of the surface of zein by forming a hydrophilic shell around the zein core and facilitating the first burst release effect [25,47]. On the other hand, the inner hydrophobic core of the nanoparticles contributes to the slower diffusion of parietin into the outer release compartment [48].

For a better understanding of the release kinetics of parietin from zein, data were fitted to three different kinetic models, as shown in Table 2. The kinetic data emphasized that the release of parietin occurs primarily by the Higuchi diffusion, as indicated by the highest regression coefficient value between the percentage of released parietin and the square root of time. Indeed, such findings approve that the zein act as a matrix through which parietin can diffuse into the exterior release compartment after solvation of the hydrophilic PEG shells by the aqueous buffer and provide a hindered release action that may sustain for several days. Moreover, the release of parietin is seemingly controlled by the various parameters affecting the polymeric matrix and drug release kinetics, including drug dissolution/diffusion rate, the rate of water uptake, the particle diameter, and the rate of matrix erosion/degradation [41]. Furthermore, zein’s hydrophobicity is supposed to augment the slowdown of water penetration into the nanoparticle’s core and hinder the diffusion of the poorly water-soluble parietin into the release compartment.

### 3.3. Generation of Singlet Molecular Oxygen ^1^O_2_

The production of singlet molecular oxygen (^1^O_2_*)* is one important property of a photosensitizer that translates into its potential photodynamic activity in solution [42]. Parietin has an absorption maximum at 434 nm; moreover, it has the fluorescence excitation and emission at 434 and 520 nm, respectively (Figure S2). Therefore, it could be excited in the blue light region. Uric acid was reported to act as an oxygen scavenger that can indirectly assess the release of singlet molecular oxygen from parietin by means of UV-Vis spectroscopy [42,49]. Additionally, it has an absorption maximum at around 291–295 nm in the ultraviolet region; hence, it remains stable even with consecutive irradiations in the blue light region [19]. Therefore, it gives a linear degradation pattern after consecutive irradiation with blue LED in the presence of parietin, as indicated in Figure S3A,B.

Figure 3C shows the quantum yield potential after consecutive irradiation of uric acid solution in PBS in the cases of free parietin, Z-PEG-, and Z-PEG-GA-loaded parietin formulations. The quantum yield was calculated according to rose Bengal as a reference photosensitizer with a value of 0.79 [43,50]. Free parietin, Z-PEG, and Z-PEG-GA showed values of 0.605, 0.539, and 0.574, respectively. Moreover, in all tested samples, the normalized uric acid absorbance correlation coefficient with respect to irradiation intervals was within acceptable regression and demonstrated no significant difference between the free and formulated parietin [51]. The quantum yield results in PBS demonstrated zein’s potential to retain the photodynamic activity of the loaded PS. The outcomes also positively affirm several reported values for diverse anthraquinone molecules [19,41].

### 3.4. Stability of Zein Nanoparticles with Serum

**Figure 4:**
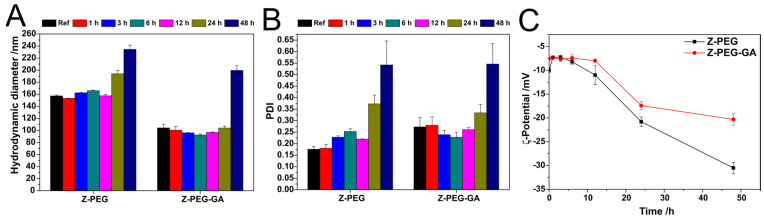
Changes in hydrodynamic diameter (**A**), PDI (**B**), and ζ-Potential (**C**) of the Z-PEG and Z-PEG-GA formulations after incubation with 10% FCS at 37 °C and 100 rpm for the course of 48 h.

The stability of the PEG-tailored zein nanoparticles was explored in bovine serum solution to assess their suitability for biological investigations. Serum proteins can adsorb to the surface of nanoparticles and affect their cellular uptake pattern or even cause nanoparticle aggregation [52]. Moreover, different coating and targeting strategies may affect protein corona development over the surface of targeted nanoparticles, which can vary with different serum sources and hence translate into the behavior of targeted nanoparticles in the biological environment [53]. Figure 4 illustrates alterations in sizes, PDI, and zeta potential of the Z-PEG and Z-PEG-GA nanoparticles at different time intervals. The nanoparticle sizes did not show any appreciable change at the first 24 h compared to the size of the reference nanoparticle, as shown in Figure 4A. However, after 48 h, a meaningful escalation in size was observed.

Similarly, the PDI values showed the same change pattern as elucidated in Figure 4B, with an obvious monodispersity at the first 24 h. In addition, the zeta potential of the PEGylated zein formulations remained unchanged at the first 12 h, as depicted in Figure 4C. However, a significant reduction in zeta potential was prominent in the next 24 and 48 h.

It is noteworthy that PEGylated platforms are less liable for aggregation when incubated with animal serum; meanwhile, a lower degree of PEGylation might induce aggregation of particles in physiological fluids [54]. Furthermore, serum proteins might adsorb to the surface of particles at physiological pH as a function of incubation time, generating an accumulation of protein corona over the nanoparticle’s surface, which induces increased size and PDI values and reduced nanoparticles’ surface potential [55]. Although PEGylated zein nanoparticles have proven their reliable stability 24 h after incubation with serum, the development of the protein corona over PEGylated zein platforms was found to have negligible influence on cellular uptake to cancer cells [56].

### 3.5. In Vitro Biological Assessment of Glycyrrhetinic Acid-Tethered Zein Nanoparticles

#### 3.5.1. Cell Viability Study

Pure GA applied to HepG2 cells was found to dose-dependently reduce cell viability, as shown in Figure S4A. The calculated IC_50_ was 106.2 µM, indicating a relative toxic effect of GA on HepG2 cells [57,58]. Alternatively, cells treated with GA-tethered zein showed that the average cell viability at the highest concentration of Z-PEG-GA was 72.2% which reflects the system’s safety on the treated cells (Figure S4B).

**Figure 5:**
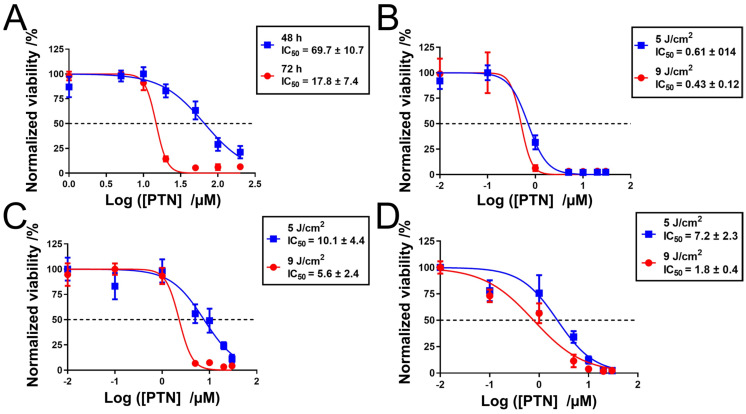
Percentage normalized viability of HepG2 cells treated for 48 and 72 h with free parietin (in 0.5% DMSO) in the dark (**A**) and after treatment with free parietin (**B**), Z-PEG (**C**), and Z-PEG-GA (**D**) incubated for 4 h and irradiated at different radiant exposures (n = 3 ± SD).

Next, the antiproliferative activity of free parietin was assessed in the absence of light to estimate its potential toxicity before irradiation. After 48 h and 72 h, parietin reduced the viability of HepG2 cell in a dose-dependent manner, as indicated in Figure 5A. The obtained IC_50_ values for free parietin were 69.7 and 17.8 µM after 48 and 72 h, respectively, which suggests that parietin had elevated toxicity with increased incubation time. These results correlate positively with our previously reported work on breast cancer cells, which showed negligible parietin toxicity in the dark after a short incubation time [19]. It is worth mentioning that pure parietin can inhibit cell proliferation in diverse cell lines as a function of incubation time without external stimulus [59]. This effect was correlated to the ability of parietin to hit some cellular targets and the induction of significant cellular events such as mitochondrial-induced apoptosis, intracellular ROS accumulation, and the activation of the caspase-3 pathway [59].

Following irradiation, the free parietin, Z-PEG, and Z-PEG-GA formulations demonstrated a dose- and energy-dependent reduction in cell viability, as shown in Figure 5B–D, with higher phototoxicity at a radiant exposure of 9 than at that of 5 J/cm^2^. The elevated phototoxicity difference at the two energy levels was prominent in the case of Z-PEG and Z-PEG-GA compared to the free parietin. Moreover, Figure 5 reveals that HepG2 cells treated with free parietin did not indicate a meaningful change in the obtained IC_50_ values for either 5 or 9 J/cm^2^. On the contrary, Z-PEG and Z-PEG-GA formulations showed a significant difference in IC_50_ values at both employed energies, an effect conveyed by the different uptake profiles of the two formulations. Compared to Z-PEG, the GA-tethered zein showed only 1.4-fold higher toxicity at 5 J/cm^2^ and about a 3-fold enhancement of parietin toxicity at 9 J/cm^2,^ as seen in Figure S4C. Therefore, we implemented the IC_50_ values at a 9 J/cm^2^ radiant exposure to conduct further biological investigations.

Cogno and coworkers reported a similar influence of free parietin on human colorectal adenocarcinoma cells (SW480), which reflected a dose-dependent toxicity of photoilluminated parietin with relatively similar toxicity at 5 and 10 J/cm^2^, which is likely the same for free parietin in our present study [60]. Additionally, Mugas et al. indicated no significant viability difference for photoactivated parietin on the cell survival of mammary carcinoma LM2 cells irradiated at 5 to 9 J/cm^2^, which illustrates the negligible effect of higher radiant exposures on the phototoxicity of free parietin in vitro [17]. Similarly, in our previous report, liposomal parietin formulations showed no significant difference between 6 and 9 J/cm^2^ on breast cancer cell lines [19]. However, despite the relative quenching of parietin after encapsulation, PEGylated zein formulations seem to be advantageous in conserving the phototoxicity of encapsulated parietin at increased radiant exposure and evading its retarded solubility behavior, an effect that was tremendously exaggerated by the GA tethering strategy.

#### 3.5.2. Cellular Uptake Study

**Figure 6:**
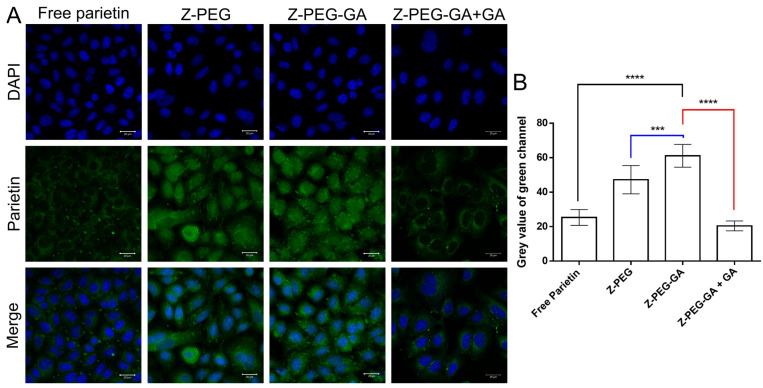
CLSM images of HepG2 cells (**A**) and the corresponding image analysis (**B**) after treatment with free parietin, Z-PEG, and Z-PEG-GA formulations for 4 h at a parietin equivalent concentration of 1.7 µM (scale bar 20 µm). Statistical significance was expressed as nonsignificant (ns), *** *p* < 0.001 and **** *p* < 0.0001.

CLSM investigations on HepG2 cells are shown in Figure 6A, demonstrating that cells incubated with Z-PEG and Z-PEG-GA formulations had higher uptake than free parietin, whose fluorescence might be quenched inside cells. Unlike encapsulated parietin, free parietin solution is readily available for cells and is not affected by release parameters that can delay the phototoxicity sequence; therefore, free parietin induced higher toxicity in the context of our study. Moreover, cells pretreated with 20 µM of free GA indicated a retardation of the uptake for the Z-PEG-GA formulation, accounting for the GA-receptor’s influence on Z-PEG-GA uptake [59]. The CLSM image analysis is illustrated in Figure 6B, showing that the Z-PEG-GA formulation obtained more significant fluorescence values than the free parietin and Z-PEG formulation, with 2.4-fold and 1.3-fold higher fluorescence, respectively. Furthermore, cells pretreated with free GA showed a 2.3-fold lower fluorescence value than the Z-PEG-GA formulation, which indicated the competitive uptake inhibition of Z-PEG-GA formulation in the presence of free GA.

The uptake of the GA-labeled molecules was reported to be a time-dependent process. Therefore, we conducted an uptake study with chemically conjugated GA with 5-aminofluorescine (GA-5AF) (Figure S5). The results indicated that after only 30 min, GA-5AF had mostly internalized into the cytoplasm of HepG2 cells. However, after 60 min, GA-5AF tended to diffuse further and colocalize with the nuclei, as depicted in Figure S6A. The mean fluorescence intensity (Figure S6B) indicated a time-dependent uptake of the GA-5AF, which was ultimately ascribed to the presence of GA receptors on the surface of HepG2 cells.

#### 3.5.3. Generation of Intracellular Reactive Oxygen (ROS)

**Figure 7:**
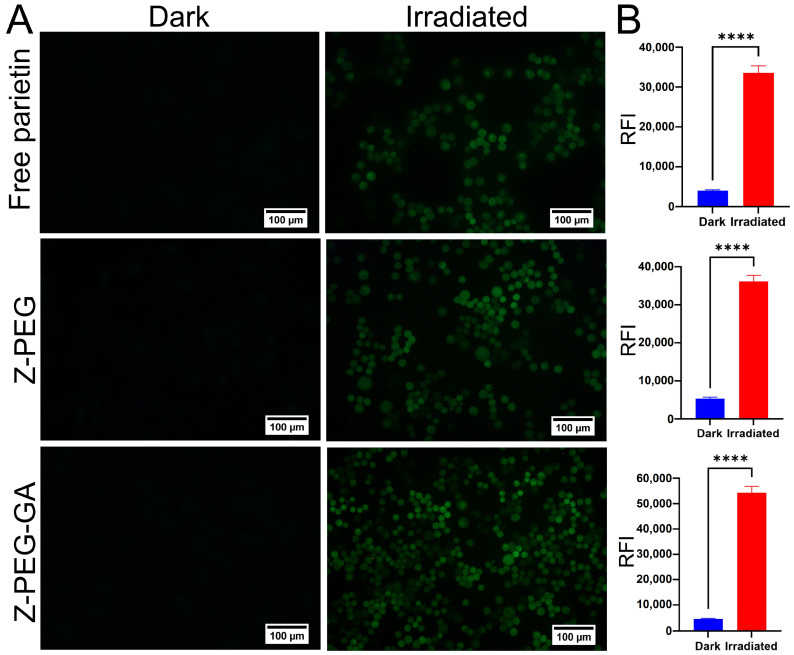
Fluorescence images (**A**) and relative fluorescence intensity graph (**B**) resulting from oxidized DCFDA following ROS generation in HepG2 cells. Cells were pretreated with free and zein-loaded parietin formulations for 4 h and irradiated with blue LED at a radiant exposure of 9 J/cm^2^. Treated cells were compared to their corresponding dark controls (scale bar 100 µm). Statistical significance was expressed as nonsignificant (ns), **** *p* < 0.0001.

The generated intracellular ROS in HepG2 cells were evaluated using H2DCFDA dye, producing green fluorescence in cells because of ROS production [61]. Relative to the dark-treated control cells, Figure 7A shows that the irradiated HepG2 cells pretreated with free and zein-loaded parietin manifested an overstated level of green fluorescence accompanied by morphological changes in cells. Nevertheless, a similar effect could not be seen in dark-treated cells.

Further ROS analysis revealed that the irradiated cells exhibited a higher fluorescence intensity than the dark-treated ones, as indicated in Figure 7B. The free and Z-PEG-formulated parietin manifested 8.3-fold and 6.8-fold increases in the produced ROS, respectively. Alternatively, the Z-PEG-GA-formulated parietin induced an 11.6-fold enhancement in intracellular ROS, indicating a significant impact of GA tethering on the intracellular ROS level of zein-formulated parietin.

#### 3.5.4. Mitochondrial Potential Disruption (ΔΨ_m_) and Release of Cytochrome C

**Figure 8:**
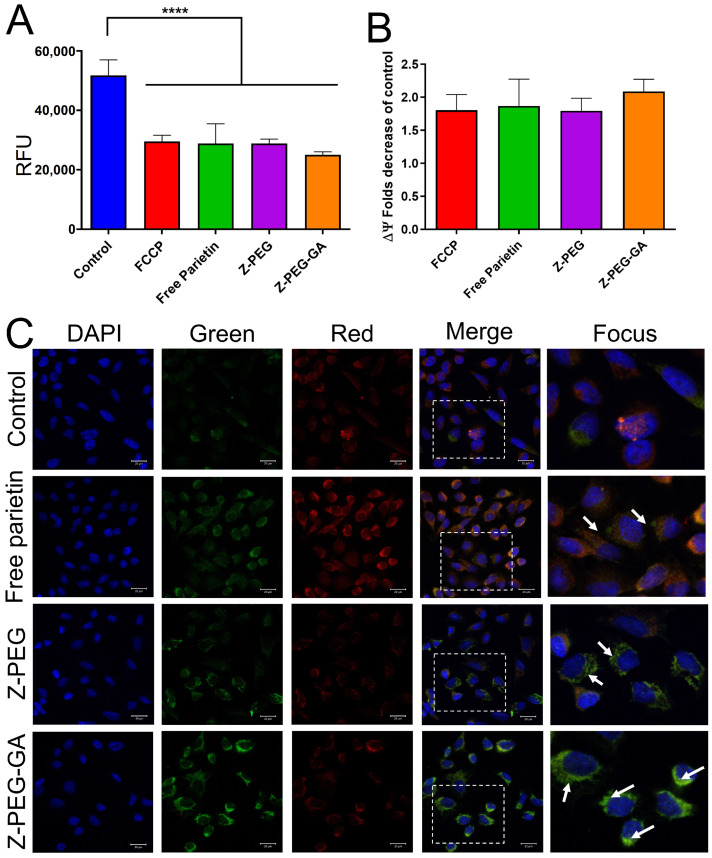
Relative TMRE fluorescence intensity (**A**), folds of mitochondrial membrane potential inhibition (**B**), and CLSM images of released cytochrome c (indicated by white arrows) (**C**) as a result of mitochondrial membrane disruption following treatment of HepG2 cells with free parietin, Z-PEG, and Z-PEG-GA for 4 h and irradiation with blue LED at a radiant exposure of 9 J/cm^2^ (scale bar 20 µm). Statistical significance was expressed as nonsignificant (ns), **** *p* < 0.0001.

Changes to the mitochondrial membrane due to parietin’s photoillumination were evaluated using TMRE as a red fluorescent label that reacts only with active mitochondria [25]. As depicted in Figure 8A, irradiation of free and zein-loaded parietin significantly reduced the relative TMRE fluorescence compared to control untreated cells. Likely, similar fluorescence patterns were obtained compared to the FCCP-treated cells (positive control). Moreover, the Z-PEG-GA formulation induced lower fluorescence than the free and Z-PEG-loaded parietin. Figure 8B indicates that the FCCP-treated cells and cells treated with the free and Z-PEG-loaded parietin formulation manifested a 1.8-fold reduction in mitochondrial membrane potential (ΔΨ_m_) compared with untreated control cells. The Z-PEG-GA provoked a more than 2-fold mitochondrial membrane potential disruption, indicating the escalated destructive effect of the photoilluminated Z-PEG-GA-loaded parietin on mitochondrial permeability.

Proapoptotic mediators tend to release upon mitochondrial disruption; therefore, we conducted an immunocytochemistry assay to estimate the potential of irradiated parietin and zein formulations to provoke cytochrome c liberation from dismantled mitochondria.

The liberation of cytochrome c is a rapid process that occurs within a short time in cells undergoing apoptosis [62]. Therefore, only the complete escape of cytochrome c could be observed by immunocytochemistry. As illustrated in Figure 8C, CLSM images of irradiated HepG2 cells pretreated with free parietin indicated a peripheral green fluorescence in the cytoplasm corresponding to the liberated cytochrome c. The Z-PEG treatment reveals abundant fluorescent dots of highly fluorescent cytochrome particles that were also obvious in the cytoplasm. On the other hand, cells pretreated with Z-PEG-GA formulation showed escalated fluorescence with some infiltration into the nucleus, as pointed out by the white arrows representing the exaggerated release pattern of cytochrome c. These observations reveal the potential of the GA-tethered zein to hasten the relief of mitochondrial cytochrome c as a key promoter in cellular apoptosis [63].

Nevertheless, none of the previous observations could be detected in dark-treated cells (Figure S7), which supports the hypothesis that only the irradiated parietin and zein formulation can provoke a mitochondrial disruption and release proapoptotic mediators after a short incubation time.

### 3.6. In Vivo Biodistribution

In the biodistribution study, we tracked the tissue distribution of GA-tethered zein nanoparticles using ICG encapsulated into Z-PEG and Z-PEG-GA using NIR real-time fluorescence imaging in mice. Each animal received an average ICG dose of 7 µg via the tail vein, where NIR signals could be recorded.

**Figure 9:**
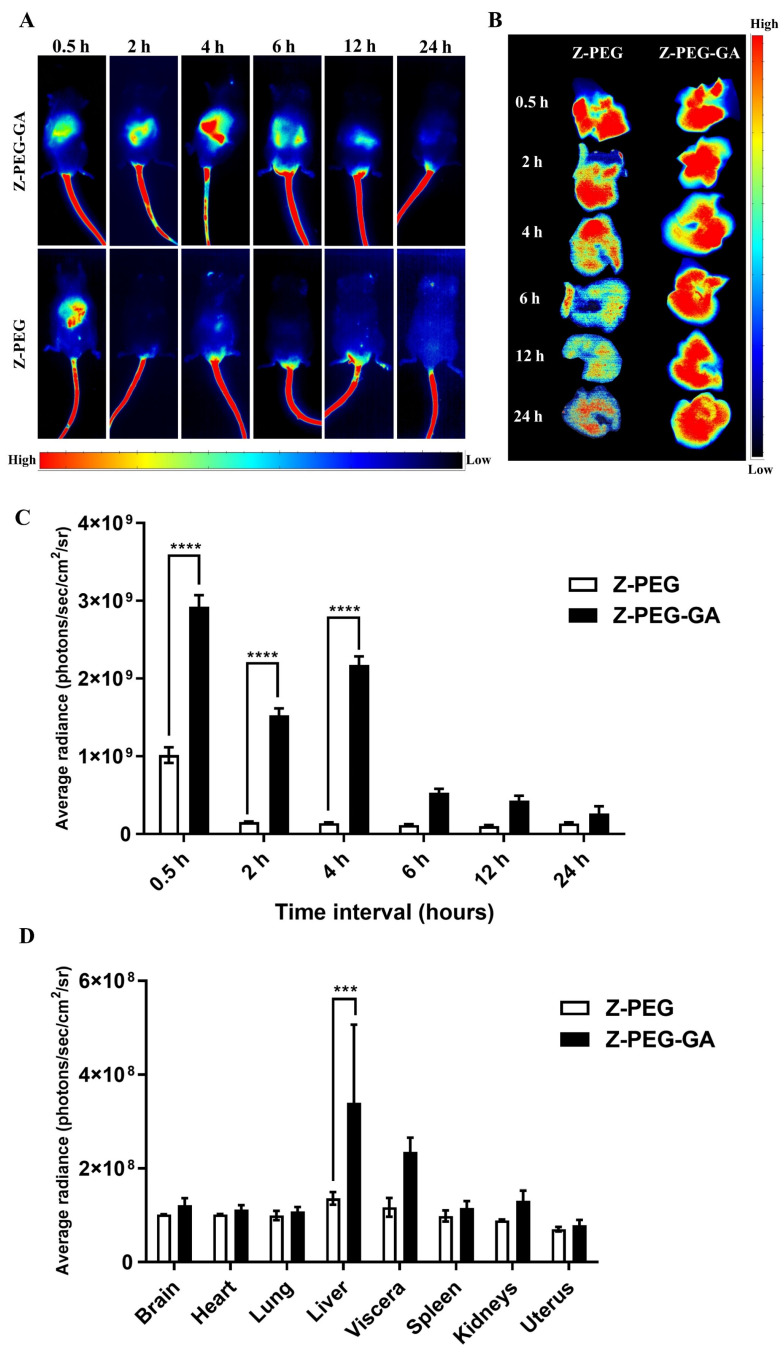
In vivo fluorescence images of the i.v injected mice with ICG-loaded Z-PEG and Z-PEG-GA formulations (**A**) and fluorescence images of mice liver isolated at 0.5, 2, 4, 6, 12, and 24 h after injection of Z-PEG and Z-PEG-GA formulations (**B**). The quantitative average NIR signals obtained from excised mice liver at different time points (**C**) and the average NIR fluorescence signals for main organs isolated from the Z-PEG- and Z-PEG-GA-injected mice after 24 h (**D**). Statistical significance was expressed as nonsignificant (ns), *** *p* < 0.001 and **** *p* < 0.0001.

As shown in Figure 9A, the fluorescence signals for the Z-PEG group can be seen 30 min after injection and start disappearing afterward. After 2 h of injection, no apparent signals were detectable up to 24 h. In contrast, the Z-PEG-GA treated group showed fluorescence signals that remained up to 12 h and were still detectable after 24 h. Ex vivo NIR imaging of the excised liver (Figure 9B) illustrated very prominent fluorescence signals in the liver of the Z-PEG-GA injected group, emphasizing that the particles are highly retained within the liver up to 24 h after injection. Comparatively, liver images of the Z-PEG group indicated fluorescence signals at the first 0.5 h, which decreased significantly after 4 h.

The quantitative analysis of the ex vivo NIR signals of the liver is shown in Figure 9C, indicating that Z-PEG-GA had a 2.8-fold higher accumulation than the Z-PEG group 30 min after injection. The difference was even more pronounced after 4 h, when Z-PEG-GA reached a 15-fold higher fluorescence intensity than the Z-PEG group. The NIR signals obtained from the main excised organs 24 h after injection are shown in Figure 9D. Z-PEG-GA signals persist in the excised animal liver for up to 24 h (1.8-fold increase compared to the Z-PEG), demonstrating the ability of GA-tethered zein to accumulate and be retained within the liver of injected animals for an extended time.

## 4. Discussion

GA-decorated zein nanoparticles were developed and evaluated by chemical and physical means. Before particle preparation, GA was successfully conjugated to zein through a PEG spacer, as confirmed by FT-IR and H^1^NMR spectroscopy. PEG conjugation to the hydrophobic zein likely reduced its hydrophobicity, particularly on the particle surface, leading to the formation of a hydrophilic shell around the zein core. This modification contributed to the initial burst release effect. In contrast, conjugating the water-insoluble GA to Z-PEG increased the hydrophobicity of both the molecules and the nanoparticles. These changes in hydrophobicity were further supported by the encapsulation efficiency of parietin, which was higher in Z-PEG-GA nanoparticles than in Z-PEG nanoparticles. Our findings showed that the developed nanoparticles were highly stable and monodisperse, with an average diameter of 94.7 nm, which conserved the biological activity of parietin. We investigated the stability of Z-PEG and Z-PEG-GA nanoparticles with bovine serum solution to take any physicochemical changes (hydrodynamic diameter, PdI, and zeta potential) under consideration when performing and evaluating all follow-up experiments (i.e., 4-h incubation in 2.6. In vitro biological investigations and 24-h incubation in 2.7. In vivo biodistribution study). Protein corona formation, which would occur when the nanoparticles were intravenously applied, is a detrimental factor that might significantly decrease nanoparticle stability and effective therapy. Our results indicate that the nanoparticles stayed stable for 24 h, minimizing the risk of stability issues influencing the in vitro and in vivo results.

Zein-parietin nanoparticles showed comparable phototoxicity towards liver cancer cells, ascribed to the unique surface tailoring. Furthermore, the cellular distribution and targeting of hepatic cancer cells were improved, as evidenced by our CLSM imaging. The pronounced phototoxicity of the Z-PEG-GA formulation toward liver cancer cells was related to the GA receptors on its surface, which can induce an enhanced parietin uptake through an active transport mechanism and escalate the probability of mitochondrial targeting [36,58,64]. Despite the high photodynamic activity of free parietin, it did not show a significant toxicity difference at the applied radiant exposures. In contrast, the Z-PEG and Z-PEG-GA revealed a noticeable difference in the toxicity of encapsulated parietin for the two employed radiant exposures, indicating a prominent effect of the designed formulations on the light-induced toxicity of parietin.

The uptake pattern of nanoparticles is one vital parameter determining the toxicity of loaded cargoes on treated cells. Our previous study assessed the different endocytosis mechanisms of PEGylated zein, including the clathrin- and non-clathrin-dependent pathways [25]. Indeed, the binding effect of GA to the surface of liver cells drove that exaggerated uptake. Such type of receptor binding was studied by Sun et al. on mammalian HepG2 cells. They proved that FITC-GA could bind on the cytomembrane in less than 30 min of incubation and gradually internalized with time to reside inside the cytoplasm and colocalize with stained nuclei within 2 h [65]. Furthermore, increasing the incubation time can count for an elevated accumulation of GA-labeled zein in the HepG2 cytoplasm, a phenomenon genuinely endowed by the presence of the protein kinase-α (GA-binding receptors) on the HepG2 surface. In contrast, our findings correlate well with several studies of nano designs developed for the GA-mediated uptake to HepG2 cells [37,66,67]. The unformulated parietin in DMSO is freely accessible to cells and can induce direct toxicity after irradiation. However, parietin might be subject to the aggregation-induced quenching (AIQ) phenomenon in its free form, which could cause reduced fluorescence inside the cells. On the other hand, away from the enhanced uptake, zein-encapsulated parietin is influenced by both encapsulation-induced quenching (EIQ) and inadequate release from the nanoparticle core, which accounts for its relatively low toxicity in the in vitro assessment [68]. Furthermore, the size of the parietin-loaded zein nanoparticles might have an impact on their activity, especially in terms of cellular uptake; however, additional studies are necessary to elucidate the influence of different particle sizes on GA-mediated uptake.

Notably, exposure to ROS triggers mitochondrial membrane permeabilization, leading to the cytosolic release of cytochrome c [69]. After that, the fragmentation of anti-apoptotic proteins in the mitochondria and endoplasmic reticulum starts to initiate apoptosis [70]. In contrast to free and Z-PEG-loaded parietin, we propose that GA tethering over the zein surface facilitates its enhanced receptor-mediated endocytosis [33,64]. In addition, external blue-LED illumination induces an elevated level of intracellular ROS that further interacts with the mitochondria. Subsequently, MMP disruption occurs because of the remodeling of the mitochondrial matrix to the condensed state and cristal unfolding, which exposes cytochrome c to the intermembrane space, facilitating its release to the cytosol, leading to the initiation of caspases and eventually cell apoptosis [71,72]. Our results agree with previously published studies, where photoactivated parietin induced intracellular ROS production and MMP disruption, leading to mitochondrial-driven apoptosis in breast cancer cells [19]. Furthermore, GA tethering has shown a significant role in the escalation of MMP disruption, which supports the fact that GA has a potential mitochondrial targeting capacity [36].

To explore the enhanced circulation time and targeting properties of zein nanoparticles in vivo, we used ICG as an IR fluorescence marker (Section 2.6). PEGylation of particles can generally cause a stealth effect and increase their circulation time in the blood stream until they reach their target in the liver. In our case, the non-targeted control nanoparticles were washed away easily from the liver. The fluorescence intensity of Z-PEG particles in the injected mice and the isolated liver was significantly lower than that of the functionalized Z-PEG-GA particles (Figure 9). Therefore, active targeting with GA can be considered a beneficial approach to enhance the residence of the conveyed cargo into the desired organ, evade accelerated clearance, and avoid the imprecise delivery of PEGylated platforms. Our findings also revealed a significant accumulation of GA-tethered zein particles in the liver of live mice with NIR signals for up to 24 h, an observation that was negligible for GA-free nanoparticles. Nevertheless, further studies are needed to assess how particle size influences the biodistribution and targeting capabilities of GA-decorated zein nanoparticles. Considering the advantages of GA as a natural targeting ligand and the biocompatibility profile of zein, we propose that GA-tethered zein might be a promising tool for the site-directed delivery of natural and hydrophobic photosensitizers to the liver.

## 5. Conclusions

In this study, we successfully fabricated GA-decorated zein nanoparticles as natural-based nanoplatforms for targeting hepatocellular carcinoma. The obtained nanoparticles were characterized for high stability, monodispersity, and direct targeting capabilities. As proof of concept, we loaded our nanoparticles with parietin, a green photosensitizer known for its hydrophobicity. Zein nanoparticles successfully solubilized parietin and retained its photodynamic activity and in vitro phototoxicity. Future studies on in vivo models are planned to confirm the better anticancer activity of our platform. We hope our findings will pave the way for synthesizing more green-based nanomaterials to tackle the toxicity challenges associated with current nanomaterials and broaden the clinical application of PDT. It can be concluded that GA-tethered zein nanoparticles are a promising tool for delivering hydrophobic photosensitizers to liver cancer cells in vitro and are anticipated to achieve a site-specific accumulation of the loaded cargo in vivo.

## Figures and Tables

**Table 1:** Hydrodynamic diameter, PDI, *ζ-*Potential, entrapment efficiency, and drug loading percentage of parietin-loaded zein formulations (n = 3 ± SD).

Formulations		Diameter ± SD (nm)	PDI ± SD	ζ-Potential ± SD (mV)	EE ± SD (%)	DL (%)
Z-PEG	blank	82.8 ± 3.7	0.19 ± 0.02	−10.7 ± 3.9		
	parietin	109.7 ± 16.9	0.24 ± 0.04	−8.3 ± 4.2	70.3 ± 11.0	1.11 ± 0.29
Z-PEG-GA	blank	94.7 ± 6.6	0.25 ± 0.04	7.1 ± 1.4		
	parietin	111.5 ± 6.3	0.19 ± 0.05	−8.1 ± 0.5	86.1 ± 7.1	1.29 ± 0.24

**Table 2:** Kinetic data of parietin released from Z-PEG and Z-PEG-GA formulation in PBS at pH 7.4 and 1% tween fitted to the zero-order, first-order, and Higuchi diffusion models.

Formulation	Parameters	Zero-Order	First Order	Higuchi Diffusion
		% Released vs. Time	Log % Released vs. Time	(% Released vs. Time ½)
Z-PEG	Slope	0.58291 ± 0.17881	0.0071 ± 0.00237	4.31347 ± 0.97279
	(r^2^)	0.63916	0.59987	0.76618
	(k)	0.63916	0.01634	4.31347
Z-PEG-GA	Slope	0.4767 ± 0.13568	0.00528 ± 0.00159	3.50077 ± 0.72689
	(r^2^)	0.67293	0.64583	0.79448
	(k)	0.4767	0.01215	3.50077

## Data Availability

The original contributions presented in this study are included in the article/supplementary material. Further inquiries can be directed to the corresponding author.

## Supplementary Materials

The following supporting information can be downloaded at https://www.mdpi.com/article/10.3390/pharmaceutics17030370/s1, Figure S1: Relative fluorescence intensity of fluorescamine dye reacted with free PEG-NH_2_ and PEG-GA. (A) Relative fluorescence intensity of 25 µM equivalent amount of PEG-NH_2_ and PEG-GA treated with fluorescamine showing 5-fold reduction in fluorescence of PEG-GA conjugate (B). Fluorescence calibration curve of pure PEG-amine reacted with fluorescamine dye at different PEG-NH_2_ concentrations (C); Figure S2: The chemical structure (A), UV/Vis (B), and fluorescence spectrum (C) of parietin in ethanol; Figure S3: UV absorption spectra (A) and corresponding absorbance fit curves (B) of 100 mM uric acid after consecutive irradiation of co-mixed uric acid with amount equivalent to 8 µg of free parietin, Z-PEG, and Z-PEG-GA formulations at different time intervals using 100 mA and 220 watt/m^2^ blue LED; Figure S4: Normalized viability of HepG2 cells treated for 24 h with free glycyrrhetinic acid (A) and cell viability after treatment with blank Z-PEG-GA formulation (B). Average IC_50_ of free parietin, Z-PEG, and Z-PEG-GA parietin-loaded formulations at 9 J/cm^2^ radiant exposure (C) (n = 3 ± SD); Figure S5: H^1^NMR spectra of 5-amino fluoresceine (A) and GA-5AF conjugate (B) in DMSO-d6; Figure S6: CLSM images of the time-resolved uptake of GA-5AF conjugate (50 µM) to HepG2 cells (A) and the mean fluorescence intensity of the green channel after 30 and 60 min of incubation (B) (scale bar 20 µm); Figure S7: CLSM immunocytochemical analysis of HepG2 cells with free parietin, Z-PEG, and Z-PEG-GA formulations kept in the dark for 4 h, showing no evidence for the release of cytochrome c.
