# Supplementary material for: Improved Photodynamic Therapy of Hepatocellular Carcinoma via Surface-Modified Protein Nanoparticles
Source: Pharmaceutics. 2025 Mar 14;17(3):370. doi: 10.3390/pharmaceutics17030370 (PMC11944767; doi:10.3390/pharmaceutics17030370)
Supplement: Supplementary file 1 [file pharmaceutics-17-00370-s001.zip › pharmaceutics-3471135-supplementary.pdf]

International Journal of *Molecular Sciences*

MDPI

Improved Photodynamic Therapy of Hepatocellular Carcinoma via Surface-Modified Protein Nanoparticles

Ahmed M. Abdelsalam [1,2], Amir Balash [3], Shaimaa M. Khedr [4], Muhammad Umair Amin [1], Konrad H. Engelhardt [1], Eduard Preis [1] and Udo Bakowsky [1,*]

1 Department of Pharmaceutics and Biopharmaceutics, University of Marburg, Robert-Koch Straße 4, 35037 Marburg, Germany; ahmed.abdelsalam@pharmazie.uni-marburg.de (A.M.A.); umairami@staff.uni-marburg.de (M.U.A.); konrad.engelhardt@pharmazie.uni-marburg.de (K.H.E.); eduard.preis@pharmazie.uni-marburg.de (E.P.)

2 Department of Pharmaceutics and Pharmaceutical Technology, Faculty of Pharmacy, Al-Azhar University, Assiut 71524, Egypt

3 Department of Pharmaceutical Chemistry, University of Marburg, Marbacher Weg 10, 35032 Marburg, Germany; amir.balash@pharmazie.uni-marburg.de

4 Pharmaceutical and Fermentation Industries Development Center (PFIDC), City of Scientific Research and Technology Applications (SRTA-City), New Borg El Arab 21111, Egypt; vetshaimaa@gmail.com

* Correspondence: ubakowsky@aol.com

S1. Materials and Methods

S1.1. Fluorescamine Assay of the Free Primary Amine (% Conjugation)

In order to indirectly determine the % of conjugated GA to the PEG molecule, a fluorescamine assay was carried out. Fluorescamine reacts only with the free amino group to give a fluorescent product that is stable for more than 4 h. A fluorescamine stock solution (0.01% w/v) was prepared in acetone. The fluorescence calibration curve of the PEG-NH2 was established by making serial dilutions (1, 5, 10, 15, 20, 25, and 30 μM) of the PEG-NH2 in 0.05 M borate buffer pH 8.5. Next, 1400 μL of each dilution was mixed with 600 μL of fluorescamine and allowed to react for 5 min before measuring the fluorescence using Perkin Elmer Fluorometer (LS 50B, USA) at 390 nm and 475 nm as the excitation and emission wavelengths, respectively. The percentage of conjugated GA (relative to reduced fluorescamine fluorescence) was estimated by dissolving a known amount of the PEG-NH-GA in borate buffer (1400 μL) and mixing with fluorescamine (600 μl). The percentage of conjugation was calculated according to the following equation:

$$\%\,Conjugation = \left(1 - \frac{Fa}{Fb}\right) x100$$

Fb is the relative fluorescence intensity of free amino-PEG before conjugation, and the **Fa** is the fluorescence intensity after conjugation with GA.

S1.2. Synthesis of GA-5AF Conjugate

GA-NHS (0.1 g, 181 mmole) was dissolved under nitrogen atmosphere in 3 mL of anhydrous DMSO. 0.63 g (181 mmole, 1 mol equivalent) of 5-aminoflourescine was added. 50 μL of triethylamine was added to the reaction mixture, and the reaction was allowed to proceed for 48 h. The reaction was ceased by dropping the mixture to ultrapure water to precipitate the GA-5AF conjugate. The conjugate was collected by filtration then redissolved in dichloromethane and washed three times with water in a separating funnel to remove excess 5AF. The organic layer was then dried with anhydrous sodium sulfate, filtered, and rotary evaporated to obtain the GA-5AF conjugate as a fluorescent green solid.

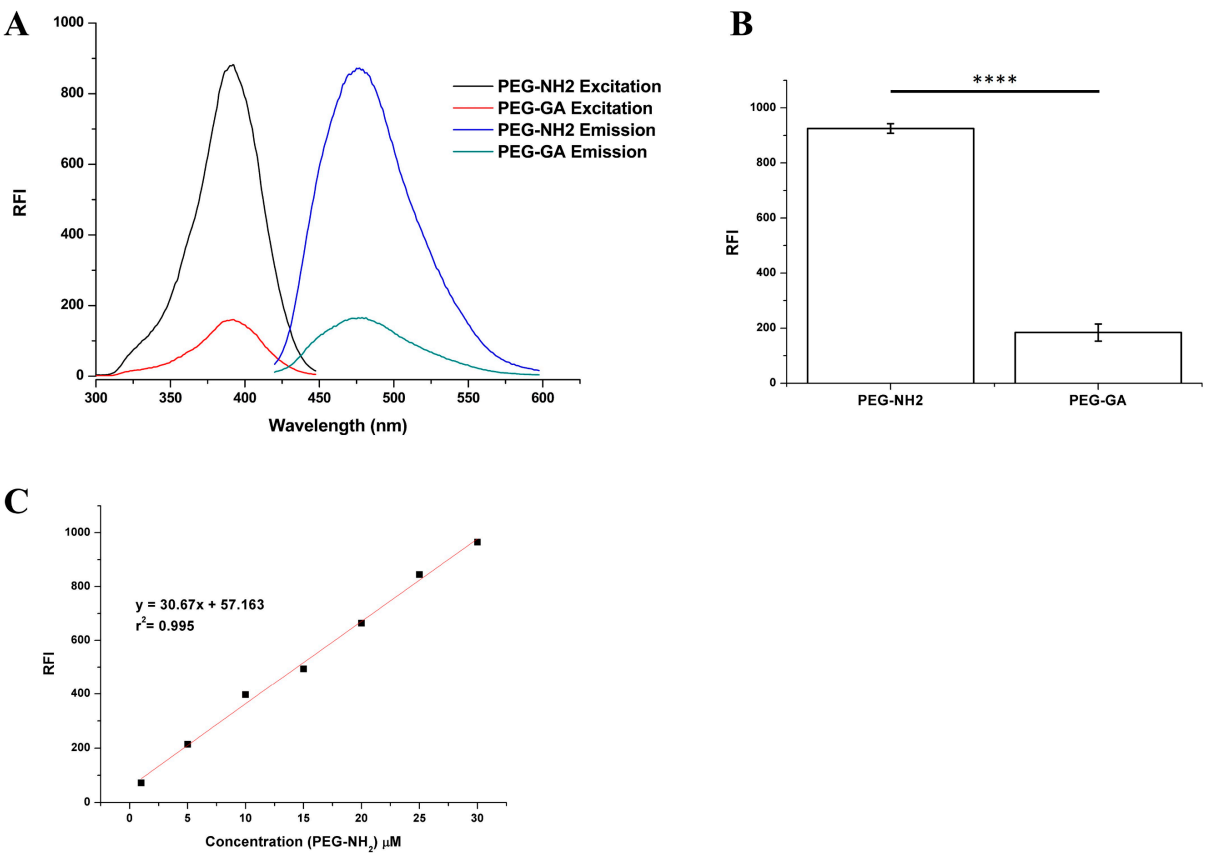

Figure S1. Relative fluorescence intensity of fluorescamine dye reacted with free $PEG-NH_2$ and PEG-GA (A) Relative fluorescence intensity of 25 µM equivalent amount of $PEG-NH_2$ and PEG-GA treated with fluorescamine showing 5 folds reduction of the fluorescence in PEG-GA conjugate (B), and the fluorescence calibration curve of pure PEG-amine reacted with fluorescamine dye at different $PEG-NH_2$ concentrations (C).

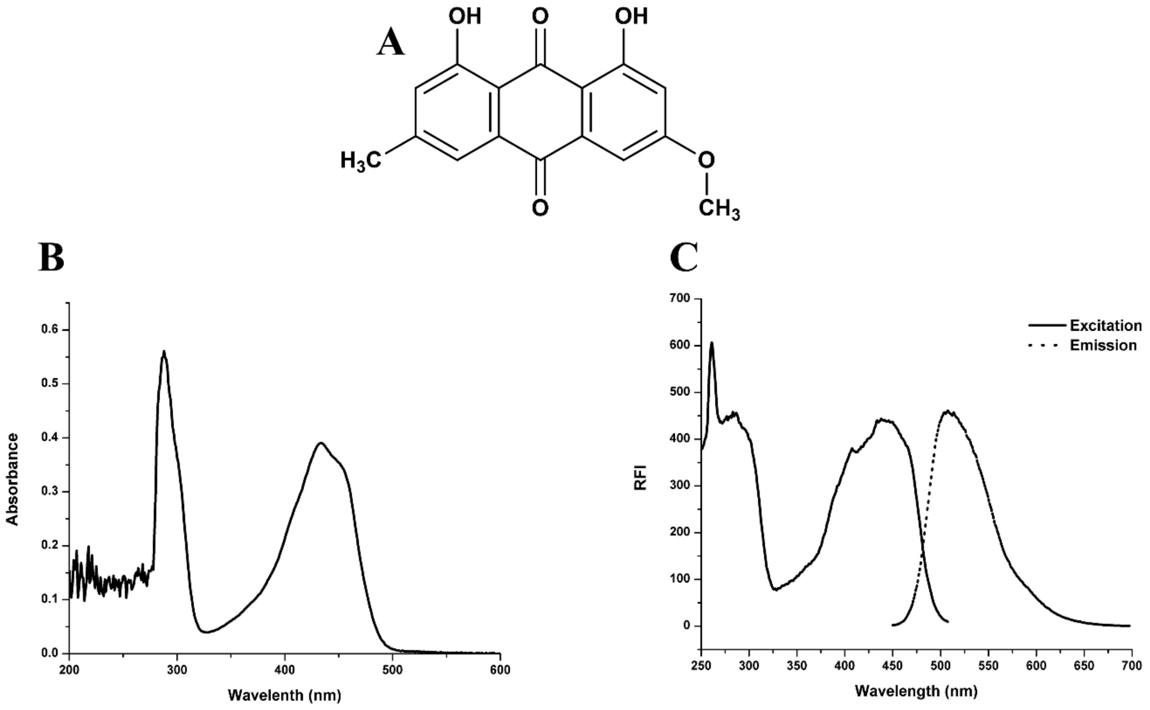

Figure S2. The chemical structure (A), UV/Vis (B), and fluorescence spectrum (C) of parietin in ethanol.

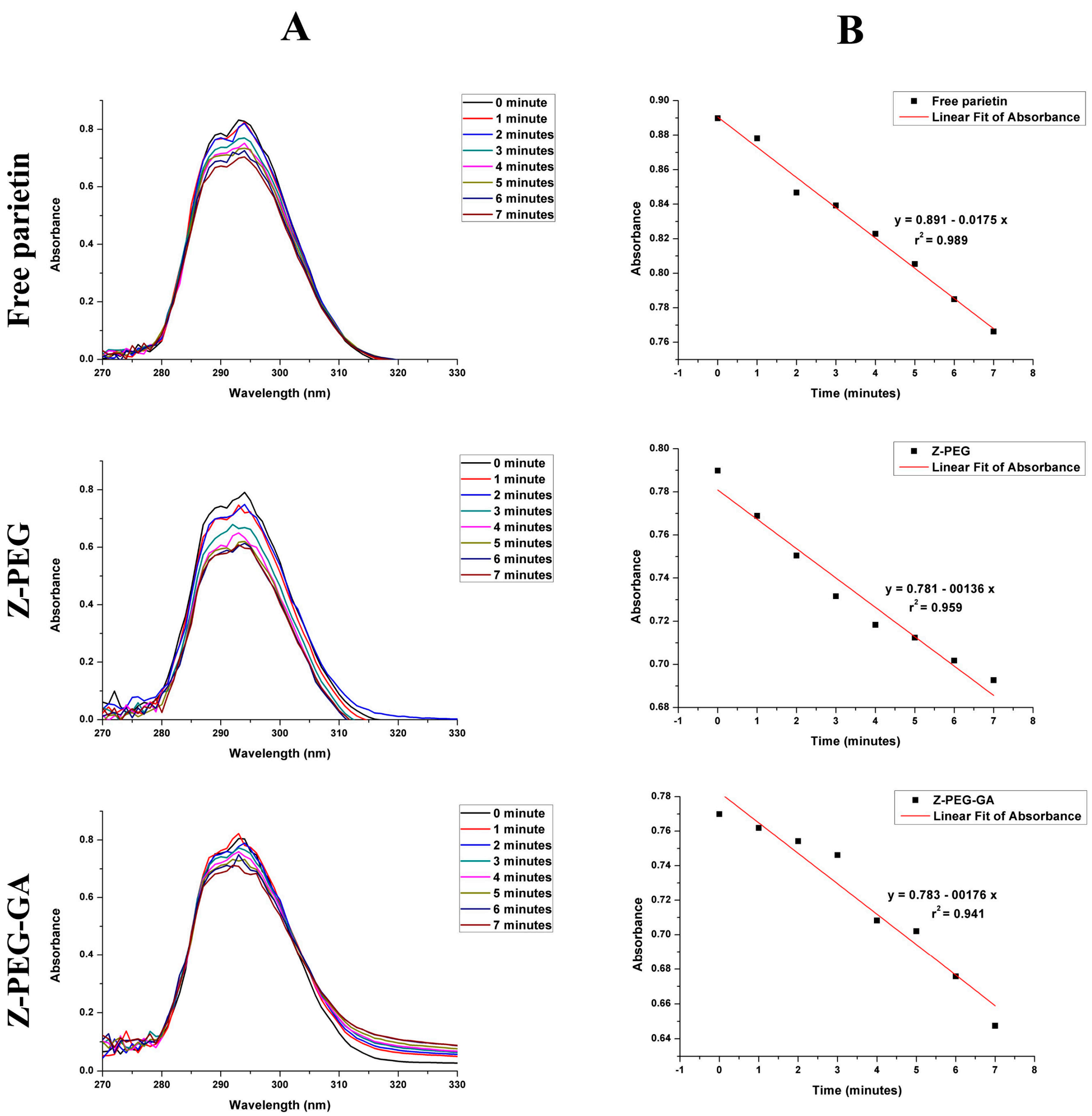

Figure S3. UV absorption spectra (A) and the corresponding absorbance fit curves (B) of 100 mM uric acid after consecutive irradiation of co-mixed uric acid with an amount equivalent to 8 μg of free parietin, Z-PEG, and Z-PEG-GA formulations at different time intervals using 100 mA and 220 watt/m^2 blue LED.

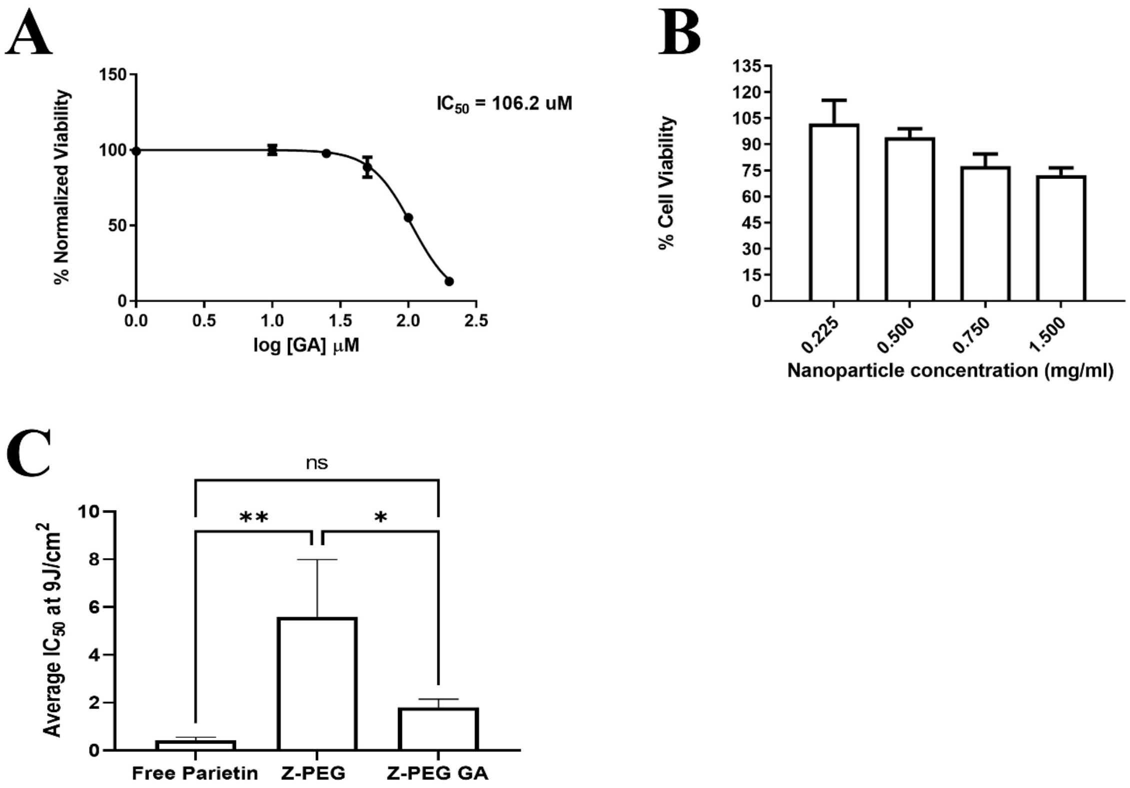

Figure S4. Normalized viability of HepG2 cells treated for 24 h with free glycyrrhetinic acid (A) and cell viability after treatment with blank Z-PEG-GA formulation (B), and the average IC_{50} of free parietin, Z-PEG, and Z-PEG-GA parietin-loaded formulations at 9 J/cm^2 radiant exposure (C), (n = 3 ± SD).

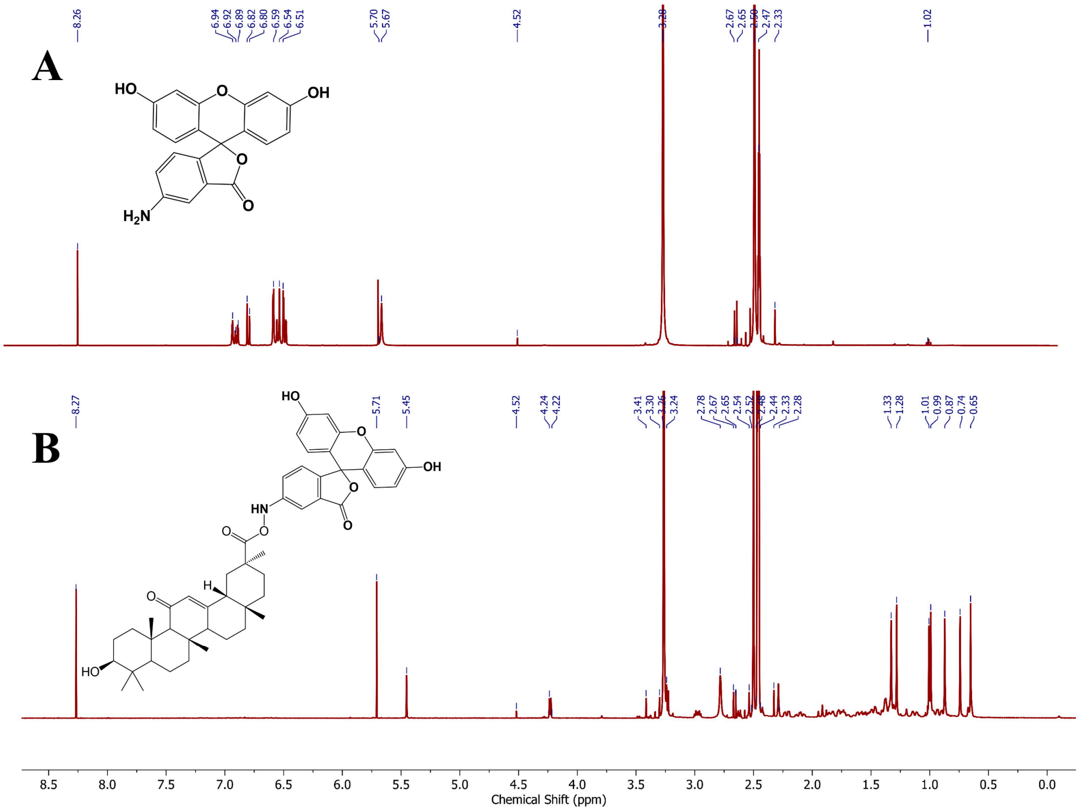

Figure S5. H[1]NMR spectra of 5-amino fluoresceine (A) and GA-5AF conjugate (B) in DMSO-d6.

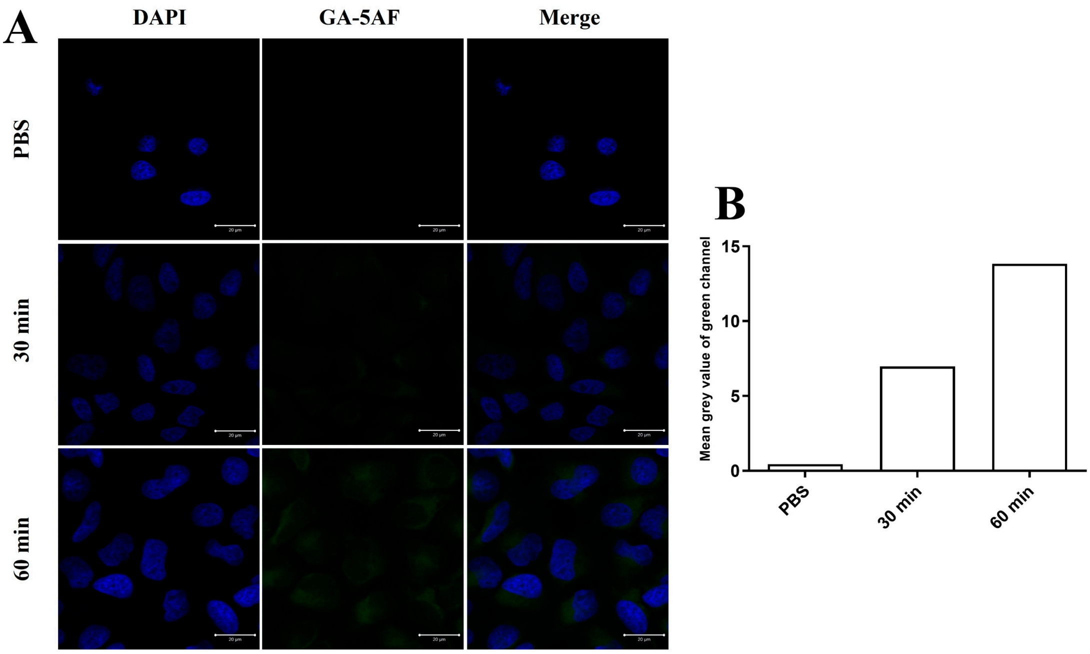

Figure S6. CLSM images of the time-resolved uptake of GA-5AF conjugate (50 μM) to HepG2 cells (A) and the mean fluorescence intensity of the green channel after 30 and 60 minutes of incubation (B). (Scale bar 20 μm).

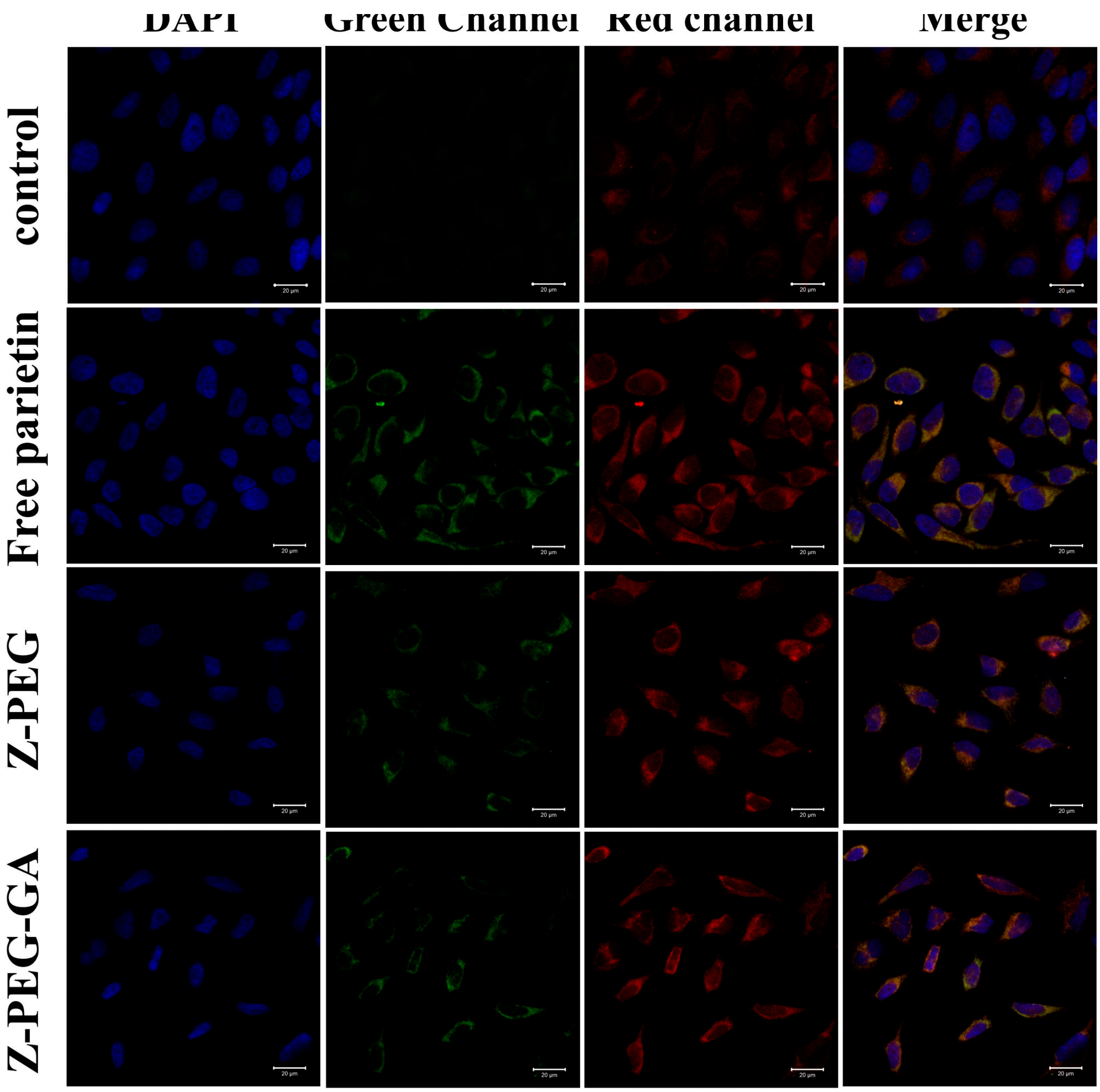

Figure S7. CLSM immunocytochemical analysis of HepG2 cells with free parietin, Z-PEG, and Z-PEG-GA formulations kept in the dark for 4 h, showing no evidence for the release of cytochrome c.

Disclaimer/Publisher's Note: The statements, opinions and data contained in all publications are solely those of the individual author(s) and contributor(s) and not of MDPI and/or the editor(s). MDPI and/or the editor(s) disclaim responsibility for any injury to people or property resulting from any ideas, methods, instructions or products referred to in the content.
